# Finite Element Analysis for Degenerative Cervical Myelopathy: Scoping Review of the Current Findings and Design Approaches, Including Recommendations on the Choice of Material Properties

**DOI:** 10.2196/48146

**Published:** 2024-03-28

**Authors:** Benjamin Davies, Samuel Schaefer, Amir Rafati Fard, Virginia Newcombe, Michael Sutcliffe

**Affiliations:** 1 Department of Medicine University of Cambridge Cambridge United Kingdom; 2 Department of Engineering University of Cambridge Cambridge United Kingdom

**Keywords:** scoping review, fine element analysis, cervical spine, spinal cord, degenerative cervical myelopathy

## Abstract

**Background:**

Degenerative cervical myelopathy (DCM) is a slow-motion spinal cord injury caused via chronic mechanical loading by spinal degenerative changes. A range of different degenerative changes can occur. Finite element analysis (FEA) can predict the distribution of mechanical stress and strain on the spinal cord to help understand the implications of any mechanical loading. One of the critical assumptions for FEA is the behavior of each anatomical element under loading (ie, its material properties).

**Objective:**

This scoping review aims to undertake a structured process to select the most appropriate material properties for use in DCM FEA. In doing so, it also provides an overview of existing modeling approaches in spinal cord disease and clinical insights into DCM.

**Methods:**

We conducted a scoping review using qualitative synthesis. Observational studies that discussed the use of FEA models involving the spinal cord in either health or disease (including DCM) were eligible for inclusion in the review. We followed the PRISMA-ScR (Preferred Reporting Items for Systematic Reviews and Meta-Analyses extension for Scoping Reviews) guidelines. The MEDLINE and Embase databases were searched to September 1, 2021. This was supplemented with citation searching to retrieve the literature used to define material properties. Duplicate title and abstract screening and data extraction were performed. The quality of evidence was appraised using the quality assessment tool we developed, adapted from the Newcastle-Ottawa Scale, and shortlisted with respect to DCM material properties, with a final recommendation provided. A qualitative synthesis of the literature is presented according to the Synthesis Without Meta-Analysis reporting guidelines.

**Results:**

A total of 60 papers were included: 41 (68%) “FEA articles” and 19 (32%) “source articles.” Most FEA articles (33/41, 80%) modeled the gray matter and white matter separately, with models typically based on tabulated data or, less frequently, a hyperelastic Ogden variant or linear elastic function. Of the 19 source articles, 14 (74%) were identified as describing the material properties of the spinal cord, of which 3 (21%) were considered most relevant to DCM. Of the 41 FEA articles, 15 (37%) focused on DCM, of which 9 (60%) focused on ossification of the posterior longitudinal ligament. Our aggregated results of DCM FEA indicate that spinal cord loading is influenced by the pattern of degenerative changes, with decompression alone (eg, laminectomy) sufficient to address this as opposed to decompression combined with other procedures (eg, laminectomy and fusion).

**Conclusions:**

FEA is a promising technique for exploring the pathobiology of DCM and informing clinical care. This review describes a structured approach to help future investigators deploy FEA for DCM. However, there are limitations to these recommendations and wider uncertainties. It is likely that these will need to be overcome to support the clinical translation of FEA to DCM.

## Introduction

Degenerative cervical myelopathy (DCM) occurs when arthritic changes to the structure of the cervical spine injure the spinal cord, causing a slowly progressive spinal cord injury (SCI) [1]. This leads to a range of different symptoms that can affect the whole body, including loss of dexterity, imbalance, altered sensation, bladder and bowel dysfunction, and pain [2]. Although DCM is estimated to affect 1 in 50 adults, <20% are estimated to receive a diagnosis. This is likely, in part, as most are only mildly affected [3,4]. Treatment is currently limited to surgery but, due to inherent risks, is reserved for those with progressive or moderate-to-severe disease [5]. Notably, <5% of patients with DCM will make a complete recovery after surgery, and instead are left with lifelong disabilities and dependence having among the lowest quality of life scores of any disease [6,7]. Consequently, this was recently estimated to cost GBP £0.7 billion (approximately US $0.9 billion) per year [8].

The etiology and pathophysiology of DCM are poorly understood [1,9]. At a macroscopic level, this is a cohort that displays progressive cervical myelopathy with degenerative changes to the structure of their cervical spine, typically causing some deformation of the spinal cord on magnetic resonance imaging (MRI), which responds to decompressive surgery. This led to the hypothesis that DCM is triggered by a chronic mechanical injury, specifically compression loading.

However, this is likely to be an oversimplification. Spinal cord compression is most commonly an incidental finding [3]; the amount of compression visualized on the MRI poorly correlates with the disease severity and does not predict the treatment response [10-12]. Moreover, many other forms of mechanical loading also occur, including stretching or shear loading. These are recognized to be capable of causing tissue injury independently [1]. For example, stretching is considered the etiology of myelopathy in tethered cord syndrome and some forms of deformity [13]. Consequently, it is more likely that the mechanical trigger in DCM is the interaction of these mechanical forces rather than one alone. As the structural changes within the spine highly vary between patients, this is likely to be a very individualized phenomenon [14]. This presents a problem for clinical practice, as conventional diagnostic tests such as MRI cannot measure mechanical stress; however, the goal of surgery is to alleviate it [12,15].

Finite element analysis (FEA) is an engineering technique that uses a computational model to derive the extent and severity of mechanical stress from an assumed loading [16]. This has frequently been applied to health care, including, to some extent, SCI and, more recently, DCM [16-18]. FEA could have important applications in DCM, both to improve our understanding of the pathobiology and to represent an individual’s injury and objectively inform surgical strategy.

To perform an FEA, a computer model incorporating the geometry, motion, and material properties of each structure must be created [17]. Geometry and motion, to a large extent, can be defined based on an individual’s clinical imaging. However, the material properties must be chosen from other sources. These choices will influence the results of the FEA. For spinal cord FEA to date, these choices have been made on a project-by-project basis, typically informed by the experience of the investigators, their interpretation and knowledge of the literature, and their specific project aims. To inform the development of FEA for DCM, we adopted an iterative approach using a scoping review methodology with the following aims:

- To describe how FEA models have been constructed with respect to spinal cord disease
- To identify and appraise the experimental literature that has informed their material property choices to make recommendations on the material properties for DCM FEA
- To aggregate the findings from studies using FEA to explore DCM.

To the best of our knowledge, this represents a unique approach to selecting the material properties for a clinical FEA model and may represent an exemplar for similar initiatives.

## Methods

A scoping review methodology was considered most appropriate to meet these objectives [19]. This scoping review was reported in accordance with the PRISMA-ScR (Preferred Reporting Items for Systematic Reviews and Meta-Analyses extension for Scoping Reviews) guidelines (Multimedia Appendix 1).

### Search Strategy

The search was conducted using a modified population, interventions, comparisons, and outcomes strategy, which states that the research question for a review must include the population, intervention, comparison, and outcome. Our research question was, “what are the current findings and design approaches for FEA in DCM?”, with the population being patients with DCM, intervention being FEA, and outcomes being current findings and design approaches. To more comprehensively guide future decisions regarding the application of FEA methods to DCM, we broadened our inclusion criteria to incorporate any study that applied FEA to the spinal cord (in either health or disease). Consequently, the search terms were designed to capture observational studies that had developed FEA models that included the spinal cord in either health or disease, including DCM (Multimedia Appendix 2). Searches were conducted from inception (February 12, 2021) to September 1, 2021, in the MEDLINE and Embase databases. Search sensitivity was evaluated using 5 papers known to meet the inclusion criteria; all papers were successfully captured [18,20-23].

### Inclusion and Exclusion Criteria

Papers were considered eligible for inclusion if they were observational studies that discussed the use of FEA models that included the spinal cord of humans or animals in either health or disease, including DCM.

Papers were excluded if they were written in a language other than English, did not use FEA models, or did not include the spinal cord in the FEA model. Furthermore, systematic reviews, scoping reviews, editorials, and abstracts were excluded.

### Study Screening and Data Extraction

Two reviewers (BMD and SS) independently performed title and abstract screening with blinding using Rayyan (Rayyan Systems Inc). A pilot screen of 100 publications was conducted to ensure concordance between reviewers. Any disagreements following unblinding were resolved by discussion between the reviewers until mutual agreement was reached. In this review, papers identified through our search strategy are termed “FEA articles”.

From the included FEA articles, the references used to justify a structure’s material properties were also screened to identify experimental studies reporting original data acquired from physical tissue tests. Studies exploring behavior computationally but including their original physical experiments, even if published elsewhere, were included. Studies that explored properties solely on a computational basis were excluded. This forward search continued within the references of a referenced study if the reference did not meet this criterion and had cited an alternative source.

Papers were retrieved for full-text screening and data extraction using a piloted pro forma. Data extracted from the papers included: author, year of publication, country, study objectives, study design (eg, human or animal study), disease of interest (if any), spinal segment (eg, cervical, thoracic, and lumbar), reference for anatomy (eg, cadaveric specimen and imaging), and details of how the FEA model was developed and validated (including the material properties of the anatomical elements).

Data extraction focused on the properties specifically referenced by the original FEA models and may not have included all the material properties discussed in the paper. To understand an investigator’s approach to model development, these were distinguished as those used to define the model a priori (ie, referenced data and the choice of material law and selected coefficients) or those used to validate the final model (if performed). However, for the purpose of selecting data to inform an FEA model, these references were aggregated and termed as “source articles” in this review.

In the absence of a standard quality assessment tool for experimental studies of biomechanics, we developed a classification to help appraise source articles that are most appropriate for a DCM FEA model [24]. This included a risk of bias assessment adapted from the Newcastle-Ottawa Scale, focusing on selection and reporting bias (Multimedia Appendix 3) [25].

### Data Analysis and Reporting

Due to significant heterogeneity between methodologies, meta-analysis was not possible, and a qualitative Synthesis Without Meta-Analysis (SWiM) was instead performed. Data were aggregated, where applicable, qualitatively, quantitatively, or using frequency statistics, as per the SWiM guidelines [26].

Given the small field size, with many papers published by single groups, citation networks were created to graphically consider which choices were made across the field and how they were informed. Using this framework and our judgment, we ranked source articles into approximate tertiles. For FEA articles that had cited top-source articles and represented the material properties using an equation, the performance of this equation was further evaluated graphically by generating stress-strain curves. These were exclusively either linear or hyperelastic. For models using a linear elastic equation, the Young modulus was used as the gradient of the stress-strain curve. For models using a hyperelastic equation, a 3×3 element cube was created using ABAQUS (Dassault Systèmes). The cube was stretched uniaxially, with no constraint applied in the orthogonal directions, linearly increasing the nominal strain in increments of 0.04 to a maximum of 0.4. The outputs of this model were then applied true stress as a function of the applied true strain. Finally, any primary clinical papers that conducted FEA for the investigation of DCM were aggregated separately and analyzed.

Data were displayed using a range of plots constructed using R Studio (version 4.0.3; Posit).

## Results

### Overall Approach of FEA Models of Spinal Cord Disease: Anatomy, Geometry, Motion, and Validation

**Figure 1:**
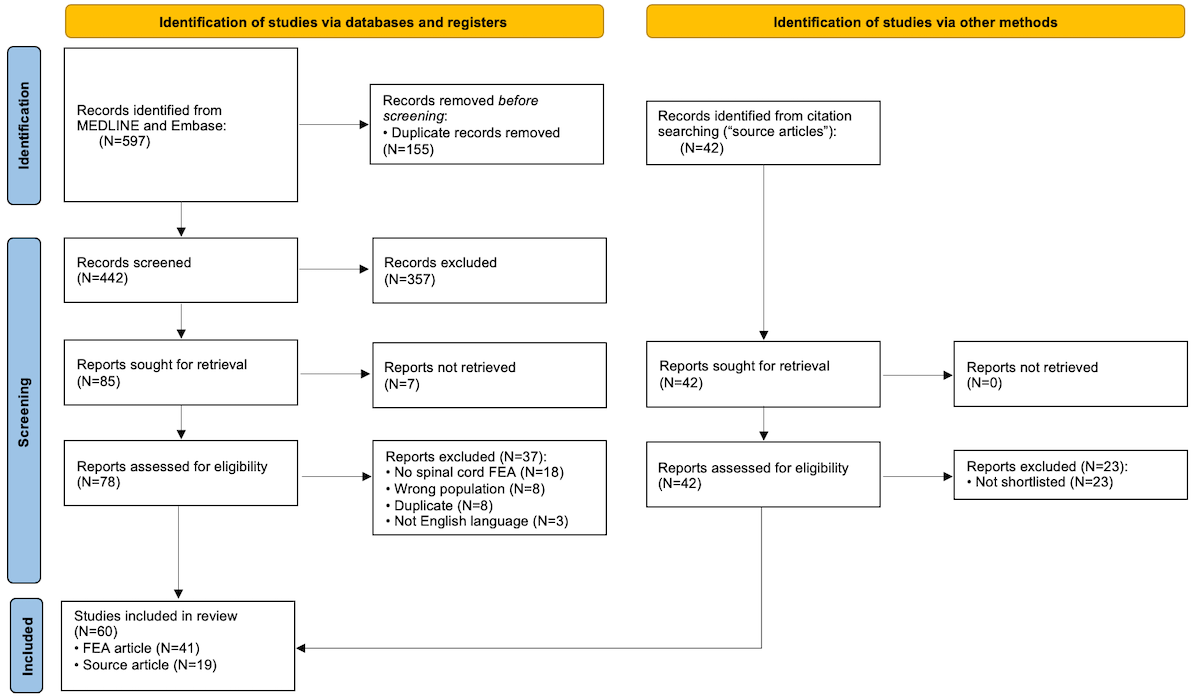
PRISMA (Preferred Reporting Items for Systematic Reviews and Meta-Analyses) flow diagram. FEA: finite element analysis.

The search returned 597 articles, of which 155 (25.9%) were duplicates (Figure 1). Following screening, 41 FEA articles were eligible for inclusion, of which 32 (78%) modeled the human spinal cord; a further 45 (7.54%) source articles were identified through citation search, of which 19 (42%) were shortlisted as suitable. Of the FEA articles, approximately half (21/41, 51%) focused on SCI [27-47]; 34% (14/41) on DCM [18,20-22,48-57]; and 5% (2/41) each on scoliosis [58,59], syringomyelia [60,61], and flexion myelopathy [62,63]. Most models (25/41, 61%) included only the spinal cord, whereas 24% (10/41) included the surrounding anatomy at multiple vertebral levels, and 17% (7/41) included the surrounding anatomy at only 1 motion segment (ie, 2 adjacent vertebrae). Physiological movement of the spine (flexion and extension) was incorporated into 17% (7/41) of the models, but none evaluated spinal cord oscillation. This was equally likely among the DCM and SCI models (Multimedia Appendix 4).

The anatomy of each model was built using a combination of imaging and cadaveric data in 27% (11/41) of the FEA articles. Typically, imaging was used for bones and cadavers for soft tissues, including the spinal cord. This included an open-source reference library called BodyWorks [64] and a review of spinal cord geometry [65]. MRI was used to define the spinal cord specifically in 20% (8/41) of the FEA articles.

For most FEA articles (33/41, 80%), the spinal cord was modeled as gray matter and white matter separately and had a defined pial layer (26/41, 63%) or was encased within the dural layer (26/41, 63%). Defined pial and dural layers were used in combination in only half of these articles (13/41, 32%). Cerebrospinal fluid (CSF) was specifically modeled in 41% (17/41) of the FEA articles, while other elements were variably included. This choice was independent of the disease and publication date (Multimedia Appendix 4). Elements were modeled using solid shell elements, unless specified differently in the Material Properties of Anatomical Elements With Recommendations for DCM FEA section.

Validation methods were specified in 63% (26/41) of the FEA articles, with 15% (6/41) using their own experiments and 9% (20/41) using literature (Multimedia Appendix 5). These references pointed to 17 articles, of which 7 (42%) provided material property data for the spinal cord in healthy circumstances and 3 (18%) in traumatic SCI circumstances. Of the remaining 17 articles, 4 (24%) described motion of the spine [66-69] and 1 (6%) described the spinal cord in flexion and extension [70]. Of the 9 articles providing information on healthy spinal cord properties, 7 (78%) were also used in other studies to inform the selection of material property. No DCM-specific validation data sets were identified.

### Material Properties of Anatomical Elements With Recommendations for DCM FEA

#### Spinal Cord

The material properties of the whole spinal cord were defined in 22% (9/41) of the FEA articles. This was rarely justified, but if so, qualified by its uncertain significance [71,72]. Typically, a hyperelastic Ogden variant (4/9, 44%) or a linear elastic (3/9, 33%) function was used.

For the remaining models, gray and white matter were modeled separately, except for the article that explored the impact of a range of white matter material properties, where the material law applied to gray matter was the same as that of white matter. The remaining 32 models were mostly based on tabulated data from the studies by Ichihara et al [72,73], and less frequently, Bilston and Thibault [74], Tunturi [75], and Ozawa et al [76]. Alternatively, a hyperelastic Ogden variant (10/41, 24%) or a linear elastic (4/41, 10%) function was used.

A total of 2 studies specifically compared different material properties with respect to a transverse contusion model of SCI. Jannesar et al [38] explored white matter properties on the basis that single constitutive models may not account for the dynamic (viscoelastic) and anisotropic properties. They identified that this could be improved by adding reinforcing functions. A second order reduced polynomial hyperelastic function combined with a quadratic reinforcing function in a 4-term Prony series performed best (0.89<*R*^2^<0.99), although this was principally in relation to the high strain rates of an SCI. Fournely et al [45] used a first-order Ogden function but varied the stiffness of the gray matter with respect to the white matter. Although this fell within the range of the validation data set, they observed differing responses to the load. When the gray matter was stiffer than the white matter, strain distribution was more diffuse and maximal within the white matter. When the stiffness was equivalent, strain was localized to the impact site. When the white matter was stiffer than the gray matter, strain was less localized, maximal within the gray matter and involved the contralateral gray matter. This was the principal factor determining behavior, ahead of other factors explored, including spinal cord diameter, curvature, and impactor angle.

A total of 2 studies similarly explored the implications of different gray and white matter material properties with respect to DCM, with similar findings discussed in the Findings From the FEA Studies of DCM section [34,50].

**Figure 2:**
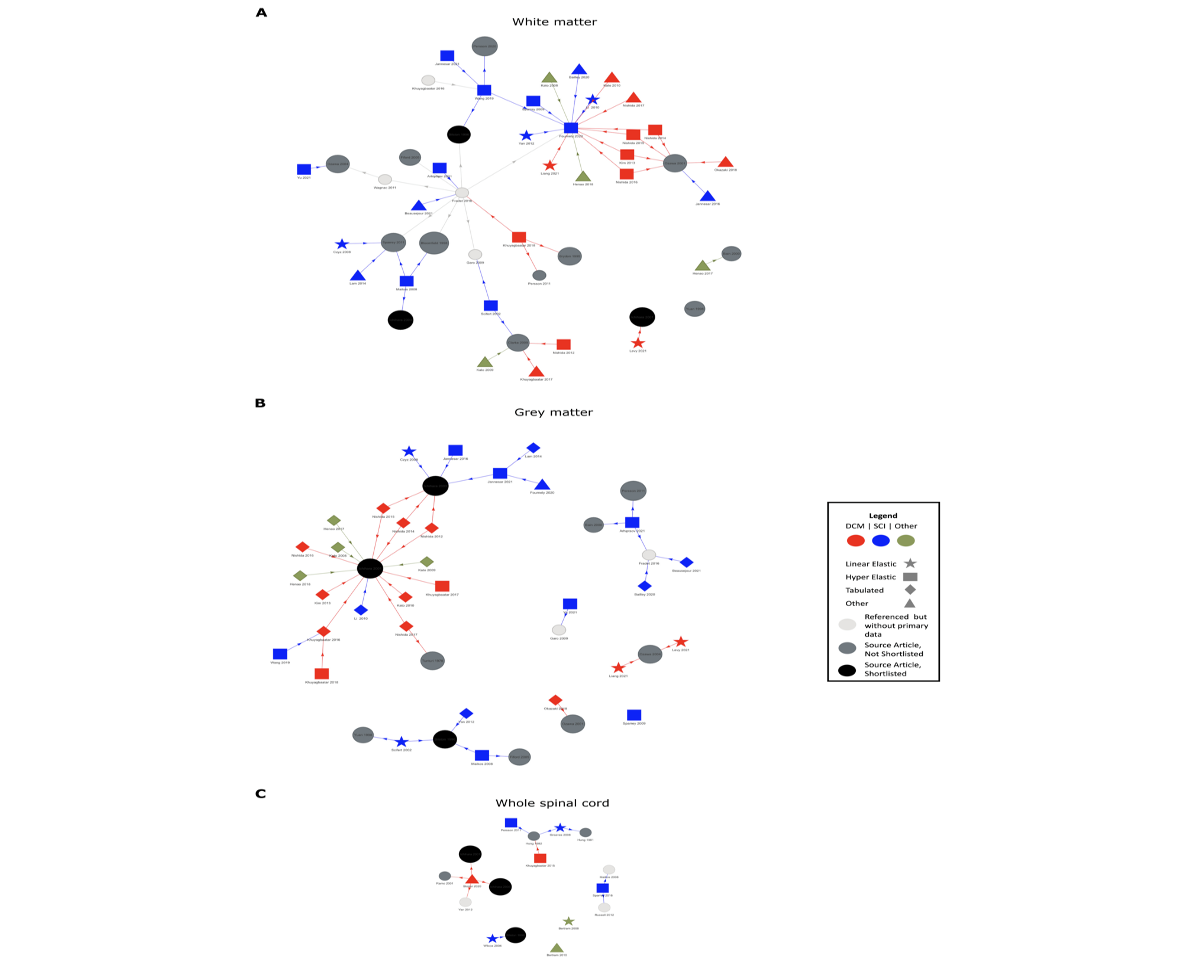
Network analysis of finite element analysis models, which is linked to a shortlisted source article, for the white matter (A) and gray matter (B) or the spinal cord as a whole (C). The original finite element analysis models are represented by their choice of material law as a star (linear elastic), square (hyperelastic), diamond (tabulated), or triangle (other) and their disease of interest as degenerative cervical myelopathy (DCM; red), spinal cord injury (SCI; blue), or other (green). These link to the primary source articles (dots). An intermediate article, that is, the one that did not include primary experimental data, is pale gray. A shortlisted source article is black. Each figure is additionally available as an interactive file; refer to Multimedia Appendix 7. The higher resolution version of this figure is available in Multimedia Appendix 8.

A total of 14 source articles were identified describing the material properties of the spinal cord or its subcomponents (Multimedia Appendix 6 [46,72-75,77-90]), of which 3 (21%) were shortlisted with relevance to an FEA for DCM [72-74]. Their interpretations varied across studies (Figure 2). The choice of material laws and values of those who directly cited the prioritized source articles and separately distinguished gray and white matter are listed in Tables 1-2. Broadly, these align with the source articles; however, there are differences across the strain range (Multimedia Appendix 6). Of these FEA articles representing material properties with an equation, studies by Jannesar et al [29] and Khuyagbaatar et al [53] were selected as these were most aligned for gray matter and white matter, respectively.

**Table 1:** Extracted material equations for the gray matter.

Study, year	Pathology	Reference	Law	Variant	E^a^ (MPa)	ν^b^	α^c^	μ^d^ (MPa)	D^e^ (MPa^−^^1^)
Jannesar et al [29], 2021^f^	SCI^g^	Ichihara et al [72], 2003	Hyperelastic	Ogden, first Order	—^h^	0.49	10.57	0.0445	*0.905^i,j^*
Khuyagbaatar et al [53], 2017	DCM^k^	Ichihara et al [73], 2001	Hyperelastic	Ogden, first order	—	*0.45*	14.7	0.0041	50.5
Jannesar et al [38], 2016	SCI	Ichihara et al [72], 2003	Hyperelastic	Ogden, first order	—	0.45	7.52	0.0306	6.77
Khuyagbaatar et al [39], 2016	SCI	Ichihara et al [73], 2001	Hyperelastic	Ogden, first order	—	*0.45*	14.7	0.0041	50.5
Czyz et al [42], 2008	SCI	Ichihara et al [72], 2003	Linear elastic	—	0.656	0.499	—	*0.2188*	—
Maikos et al [43], 2008	SCI	Bilston and Thibault [74], 1996	Hyperelastic	Ogden, first order	—	0.45	4.7	0.0320	6.47
Scifert et al [44], 2002	SCI	Bilston and Thibault [74], 1996	Linear elastic	—	0.0667	0.499	—	*0.0222*	—

^a^E: Young modulus.

^b^ν: Poisson ratio. Where missing, the value of ν was assumed to be 0.45.

^c^α: material exponent parameter.

^d^μ: ground shear hyperelastic modulus.

^e^D: compressibility constant.

^f^The single preferred source of the authors based on modeling (Multimedia Appendix 6), where a range of equations were put forward.

^g^SCI: spinal cord injury.

^h^Not available.

^i^Denotes a suspected error in original text and input value given.

^j^Values in italics are input based on the identity for isotropic materials, D=3(1-2ν)/(μ{1+ν}), and for linear elastic, μ=E/(2{1+ν}).

^k^DCM: degenerative cervical myelopathy.

**Table 2:** Extracted material equations for the white matter.

Study, year	Pathology	Reference	Law	Variant	E^a^ (MPa)	ν^b^	α^c^	μ^d^ (MPa)	D^e^ (MPa^−^^1^)
Liang et al [48], 2021	DCM^f^	Ichihara et al [73], 2001	Linear elastic	—^g^	4.2	0.45	—	*1.4483^h^*	—
Khuyagbaatar et al [52], 2017	DCM	Ichihara et al [73], 2001	Hyperelastic	Ogden, first order	—	*0.45*	12.5	0.0040	51.7
Khuyagbaatar et al [39], 2016	SCI^i^	Ichihara et al [73], 2001	Hyperelastic	Ogden, first order	—	*0.45*	12.5	0.0040	51.7
Czyz et al [42], 2008	SCI	Ichihara et al [72], 2003	Linear elastic	—	0.277	0.499	—	*0.0924*	—
Maikos et al [43], 2008	SCI	Bilston and Thibault [74], 1996	Hyperelastic	Ogden, first order	—	0.45	4.7	0.0320	6.47
Scifert et al [44], 2002	SCI	Bilston and Thibault [74], 1996	Linear elastic	—	0.0667	0.499	—	*0.0222*	—

^a^E: Young modulus.

^b^ν: Poisson ratio. Where missing, the value of ν was assumed to be 0.45.

^c^α: material exponent parameter.

^d^μ: ground shear hyperelastic modulus.

^e^D: compressibility constant.

^f^DCM: degenerative cervical myelopathy.

^g^Not available.

^h^Values in italics are input based on the identity for isotropic materials, D=3(1-2ν)/(μ{1+ν}), and for linear elastic, μ=E/(2{1+ν}).

^i^SCI: spinal cord injury.

#### Pia

Of the 26 FEA articles with defined pia, 14 (54%) used a linear elastic function, 9 (21%) did not report their method, and 2 (5%) used a hyperelastic Ogden variant function. The remaining study (1/26, 4%) used tabulated data from the study by Ichihara et al [73].

A total of 4 source articles were identified for the pia (Multimedia Appendix 6), of which 2 (50%) were shortlisted as suitable [75,77]. The choice of material laws and the values of those who directly cited these shortlisted source articles are listed in Table 3. These equations have differences in how they represent the source article (Multimedia Appendix 6). Of the FEA articles representing material properties with an equation, the study by Jannesar et al [38] was selected as the most preferred.

**Table 3:** Extracted material equations for the pia.

Study, year	Pathology	Reference	Law	Variant	E^a^ (MPa)	ν^b^	α^c^	μ^d^ (MPa)	D^e^ (MPa^−^^1^)
Henao et al [58,59], 2017	Other	Tunturi [75], 1978	Linear elastic	—^f^	100	0.4	—	*35.71^g^*	—
Nishida et al [91], 2016	DCM^h^	Tunturi [75], 1978	—	—	—	—	—	—	—
Nishida et al [54], 2015	DCM	Tunturi [75], 1978	—	—	—	—	—	—	—
Nishida et al [55], 2014	DCM	Tunturi [75], 1978	—	—	—	—	—	—	—
Nishida et al [22], 2012	DCM	Tunturi [75], 1978	—	—	—	—	—	—	—
Henao et al [58], 2018	Other	Tunturi [75], 1978	Linear elastic	—	100	0.4	—	*35.71*	—
Kato et al [56], 2010	DCM	Tunturi [75], 1978	—	—	—	—	—	—	—
Kato et al [62], 2008	Other	Tunturi [75], 1978	—	—	—	—	—	—	—
Kato et al [63], 2009	Other	Tunturi [75], 1978	—	—	—	—	—	—	—
Jannesar et al [38], 2016^i^	SCI	Kimpara et al [77], 2006	Linear elastic	—	39.3	0.3	—	*15.12*	—

^a^E: Young modulus.

^b^ν: Poisson ratio. Where missing, the value of ν was assumed to be 0.45.

^c^α: material exponent parameter.

^d^μ: ground shear hyperelastic modulus.

^e^D: compressibility constant.

^f^Not available.

^g^Values in italics are input based on the identity for isotropic materials, D=3(1-2ν)/(μ{1+ν}), and for linear elastic, μ=E/(2{1+ν}).

^h^DCM: degenerative cervical myelopathy.

^i^The single preferred source of the authors based on modelling (Multimedia Appendix 6).

#### Dura

Of the 26 models with defined dura, 18 (69%) used a linear elastic function, 5 (19%) used a hyperelastic Ogden variant, and 3 (12%) did not report their method.

Persson et al [46] compared the performance of a linear and hyperelastic function, which is summarized in the following CSF section.

A total of 9 source articles were referenced (Multimedia Appendix 6), of which 4 (44%) were shortlisted [78-81]. The choice of material laws and values of those who directly cited these prioritized source articles are listed in Table 4. These equations have differences in how they represent the source article (Multimedia Appendix 6). Of the FEA articles representing material properties with an equation, the study by Sparrey et al [33] was selected as preferred.

**Table 4:** Extracted material equations for the dura.

Study, year	Pathology	Reference	Law	Variant	E^a^ (MPa)	ν^b^	α^c^	μ^d^ (MPa)	D^e^ (MPa^−^^1^)
Stoner et al [20], 2020	DCM^f^	Persson et al [92], 2020	Linear Elastic	—^g^	5	0.45	—	*1.72* ^h^	—
Khuyagbaatar et al [49], 2018	DCM	Persson et al [92], 2020	Linear Elastic	—	80	0.49	—	*26.85*	—
Henao et al [58,59], 2017	Other	Wilcox et al [47], 2004	Linear Elastic	—	231	0.45	—	*79.66*	—
Khuyagbaatar et al [52], 2017	DCM	Persson et al [92], 2020	Linear Elastic	—	80	0.49	—	*26.85*	—
Sparrey et al [33], 2016^i^	SCI	Hong et al [78], 2011 and Zarzur et al [79], 1996	Hyper-elastic	Ogden,1st Order	—	0.45	16.2	1.205	0.172
Yan et al [36], 2012	SCI	Wilcox et al [47], 2004	Linear Elastic	—	142	0.45	—	*48.97*	—
Henao et al [58], 2018	Other	Wilcox et al [47], 2004	Linear Elastic	—	231	0.45	—	*79.66*	—
Khuyagbaatar et al [39], 2016	SCI	Persson et al [92], 2020	Linear Elastic	—	80	0.49	—	*26.85*	—
Khuyagbaatar et al [57], 2015	DCM	Persson et al [92], 2020	Linear Elastic	—	80	0.49	—	*26.85*	—
Khuyagbaatar et al [57], 2015	SCI	Persson et al [92], 2020	Linear Elastic	—	80	0.49	—	*26.85*	—
Czyz et al [42], 2008	SCI	Wilcox et al [47], 2004	Linear Elastic	—	142	0.45	—	*48.97*	—
Persson et al [46], 2011	SCI	Persson et al [92], 2020	Linear Elastic	—	80	0.49	—	*26.85*	—
Wilcox et al [47], 2004	SCI	Wilcox et al [47], 2004	Anisotropic Elastic	—	Young modulus in the radial direction=142, Young modulus in the circumferential direction=142, Young modulus in the longitudinal direction=0.7	—	—	—	—

^a^E: Young modulus.

^b^ν: Poisson ratio. Where missing, the value of ν was assumed to be 0.45.

^c^α: material exponent parameter.

^d^μ: ground shear hyperelastic modulus.

^e^D: compressibility constant.

^f^DCM: degenerative cervical myelopathy.

^g^Not available.

^h^Values in italics are input based on the identity for isotropic materials, D=3(1-2ν)/(μ{1+ν}), and for linear elastic, μ=E/(2{1+ν}).

^i^The single preferred source of the authors based on modelling (Multimedia Appendix 6).

#### Dentate Ligament

Of the 13 FEA articles that included the dentate ligament, 12 (92%) used a linear elastic function and 1 (8%) used tabulated data. Typically, these were modeled using shell elements (6/13, 46%) with geometric properties, but 8% (1/13) used link elements and 15% (2/13) used spring elements.

A total of 2 source articles were referenced (Multimedia Appendix 6), of which both were shortlisted [75,82]. The choice of material laws and values of those who directly cited these prioritized source articles are listed in Table 5.

**Table 5:** Extracted material equations for the dentate.

Study, year	Pathology	Reference	Law	Variant	E^a^ (MPa)	ν^b^	α^c^	μ^d^ (MPa)	D^e^ (MPa^−^^1^)
Henao et al [58,59], 2017	Other	Tunturi [75], 1978	Linear elastic	—^f^	100	0.4	—	*35.7* ^g^	—
Henao et al [58], 2018	Other	Tunturi [75], 1978	Linear elastic	—	100	0.4	—	*35.7*	—
Greaves et al [41], 2008	SCI^h^	Tunturi [75], 1978	Linear elastic	—	5.8	—	—	*2.0*	—
Czyz et al [42], 2008	SCI	Tunturi [75], 1978	Linear elastic	—	100	0.3	—	*38.5*	—

^a^E: Young modulus.

^b^ν: Poisson ratio. Where missing, the value of ν was assumed to be 0.45.

^c^α: material exponent parameter.

^d^μ: ground shear hyperelastic modulus.

^e^D: compressibility constant.

^f^Not available.

^g^Values in italics are input based on the identity for isotropic materials, D=3(1-2ν)/(μ{1+ν}), and for linear elastic, μ=E/(2{1+ν}).

^h^SCI: spinal cord injury.

#### Cerebrospinal Fluid

Of the 17 models that included CSF, 8 (47%) modeled it as a Newtonian fluid. Alternatives included modeling CSF as a pressurized fluid cavity (1/17, 6%), modeling it as a polynomial equation of state (1/17, 6%), modeling it as smoothed particular hydrodynamics (1/17, 6%), using a hyperelastic Mooney-Rivlin model (3/17, 18%), or using a linear elastic equation (1/17, 6%).

Persson et al [46] and Jones et al [93] specifically explored the implications of including a CSF cavity, with or without the dura. To measure cord deformation, Persson et al [46] used an FEA model with reference to a transverse bovine impaction model of SCI, whereas Jones et al [93] performed their own bovine and surrogate cord experiments. They observed that the presence of CSF reduced stress and strain (Persson et al [46]) on the spinal cord and deformation (Jones et al [93]) in the spinal cord. Persson et al [46] demonstrated this was through a greater longitudinal distribution, particularly when the dura was included and modeled using a hyperelastic Ogden (as opposed to linear elastic) function. Furthermore, Persson et al [46] observed that cord deformation occurred upon contact with the dura (before the CSF between the spinal cord and the dura was redistributed). Jones et al [93] observed that the inclusion of the dura only changed behavior if CSF was also included.

Furthermore, Arhiptsov and Marom [31] explored CSF pressure, alongside the presence or absence of epidural fat, using a computational contusion model of SCI based on a thoracic burst fracture. Both CSF and epidural fat were modeled using smoothed particular hydrodynamics. In a model without epidural fat, spinal cord stress and strain increased with increasing CSF pressure. However, in the model with epidural fat, spinal cord stress and strain decreased with increasing CSF pressure.

A total of 5 source articles were referenced (Multimedia Appendix 6), of which 3 (60%) were shortlisted [46,83,84]. The choice of material laws and values of those who directly cited these prioritized source articles are listed in Table 6.

**Table 6:** Extracted material equations for the cerebrospinal fluid.

Study, year	Pathology	Reference	Law	Viscosity (Pa/s)	Density (kg/m^3^)
Khuyagbaatar et al [52], 2017	DCM^a^	Bloomfield et al [83], 1998	Newtonian Fluid	0.001	—^b^
Arhiptsov [31], 2021	SCI^c^	Persson et al [46], 2011	Polynomial Equation of State	—	—
Khuyagbaatar et al [39], 2016	DCM	Bloomfield et al [83], 1998, Brydon et al [84], 1995	Newtonian Fluid	0.001	—
Khuyagbaatar et al [39], 2016	SCI	Bloomfield et al [83], 1998, Brydon et al [84], 1995	Newtonian Fluid	0.001	1000
Khuyagbaatar et al [57], 2015	DCM	Bloomfield et al [83], 1998, Brydon et al [84], 1995	Newtonian Fluid	0.001	—
Khuyagbaatar et al [57], 2015	SCI	Bloomfield et al [83], 1998, Brydon et al [84], 1995	Newtonian Fluid	0.001	—
Persson et al [46], 2011	SCI	Bloomfield et al [83], 1998	Newtonian Fluid	0.001	—

^a^DCM: degenerative cervical myelopathy.

^b^Not available.

^c^SCI: spinal cord injury.

#### Posterior Longitudinal Ligament and Ligamentum Flavum

The analysis focused on the posterior longitudinal ligament and ligamentum flavum, given their specific involvement in the pathobiology of DCM. In all 6 instances included, they were included together and modeled in the same manner: using piecewise linear plasticity (2/6, 33%), linear elastic function (2/6, 33%), hyperelastic Ogden variant (1/6, 17%), or tabulated data (1/6, 17%).

A total of 6 source articles were referenced (Multimedia Appendix 6), of which 3 (50%) were shortlisted [85-87]. The choice of material laws and values of those who directly cited these prioritized source articles are listed in Tables 7 and 8.

**Table 7:** Extracted material equations for the ligamentum flavum.

Study, year	Pathology	Reference	Law	Variant	E^a^ (MPa)	ν^b^	α^c^	μ^d^ (MPa)	D^e^ (MPa^−^^1^)
Greaves et al [41], 2008	SCI^f^	Yoganandan et al 1989 and 2000 [86,87]	Linear elastic	—^g^	3.8	—	—	*1.3* ^h^	—

^a^E: Young modulus.

^b^ν: Poisson ratio. Where missing, the value of ν was assumed to be 0.45.

^c^α: material exponent parameter.

^d^μ: ground shear hyperelastic modulus.

^e^D: compressibility constant.

^f^SCI: spinal cord injury.

^g^Not available.

^h^Values in italics are input based on the identity for isotropic materials, D=3(1-2ν)/(μ{1+ν}), and for linear elastic, μ=E/(2{1+ν}).

**Table 8:** Extracted material equations for the posterior longitudinal ligament.

Study, year	Pathology	Reference	Law	Variant	E^a^ (MPa)	ν^b^	α^c^	μ^d^ (MPa)	D^e^ (MPa^−^^1^)
Greaves et al [41], 2008	SCI^f^	Przybylski et al [85], 1996 and Yoganandan 1989 and 2000 [86,87]	Linear elastic	—^g^	35.7	—	—	*12.3* ^h^	—

^a^E: Young modulus.

^b^ν: Poisson ratio. Where missing, the value of ν was assumed to be 0.45.

^c^α: material exponent parameter.

^d^μ: ground shear hyperelastic modulus.

^e^D: compressibility constant.

^f^SCI: spinal cord injury.

^g^Not available.

^h^Values in italics are input based on the identity for isotropic materials, D=3(1-2ν)/(μ{1+ν}), and for linear elastic, μ=E/(2{1+ν}).

#### Spinal Roots

A total of 7 models included spinal nerve roots, of which 2 (29%) distinguished between the intradural and extradural components. These 2 models specifically explored the nature of C5 palsy in relation to surgery for DCM [49,57]. Nerve roots were all modeled with spring elements, either as a spring (5/7, 71%) or with a linear elastic equation (2/7, 29%).

A total of 2 source articles of equivalent quality were referenced (Multimedia Appendix 6) [88,89]. The choice of material laws and values of those who directly cited these prioritized source articles are listed in Table 9.

**Table 9:** Extracted material equations for the nerve roots.

Study, year	Pathology	Reference	Law	E^a^ (MPa)	ν^b^	Spring constant	Mass (g)
Lévy et al [18], 2021	DCM^c^	Kulkarni [88], 2007	Spring	—^d^	—	0.133	0.1
Khuyagbaatar et al [49], 2018	DCM	Singh [89], 2005	Linear Elastic	1.3	0.3	—	—
Henao et al [58,59], 2017	Other	Kulkarni [88], 2007	Spring	—	—	0.133	—
Khuyagbaatar et al [52], 2017	DCM	Singh [89], 2005	Linear Elastic	1.3	0.3	—	—
Henao et al [58], 2018	Other	Kulkarni [88], 2007	Spring	—	—	0.133	—

^a^E: Young modulus.

^b^ν: Poisson ratio; where missing, ν was assumed to be 0.45. For Kulkarni et al [88], the unit is uncertain, with a range of different units referenced across its citations.

^c^DCM: degenerative cervical myelopathy.

^d^Not available.

#### Other Elements

Other elements included in some models were bone (14/41, 34%); intervertebral disks (IVDs; 13/41, 31%); and the remaining spinal ligaments, such as the anterior longitudinal or interspinous ligament.

The bone was generally modeled as a rigid body (8/14, 57%). Of the 8 models, 3 (21%) subdivided the vertebrae into anatomical subcomponents (eg, body, laminae, and spinous process), and 5 (36%) distinguished between cortical and cancellous bone, of which 3 (60%) applied an equation just to the cortical bone (linear elastic in all cases) and 2 (40%) applied a Johnson-Cook or plastic kinematic equation. We found no eligible source articles using our search process.

The IVD were modeled as a single entity in 54% (7/13) of the papers, typically as a rigid body (5/7, 71%) or using a linear elastic equation (2/7, 29%). Alternatively, they were modeled separately as nucleus pulposus and annulus fibrosus. Techniques for the nucleus pulposus included a Mooney-Rivlin model (3/6, 50%), Ogden second-order variant (1/6, 17%), and fluid elements (2/6, 33%). The annulus fibrosus included a Mooney-Rivlin model (2/6, 33%), Ogden second-order variant (1/6, 17%), Ogden third-order variant (1/6, 17%), and linear elastic equation (2/6, 33%).

A total of 3 source articles were found for IVD, and 1 was shortlisted (Multimedia Appendix 6) [90]. The choice of material laws and values of those who directly cited these prioritized source articles are listed in Table 10.

**Table 10:** Extracted material equations for the intervertebral disc.

Study, year	Pathology	Reference	Law	Variant	E^a^ (MPa)	ν^b^	α^c^	μ^d^ (MPa)	D^e^ (MPa^−^^1^)
Greaves et al [41], 2008	SCI^f^	Spilker et al [90], 1986	Linear elastic	—^g^	3.4	—	—	*1.2* ^h^	—

^a^E: Young modulus.

^b^ν: Poisson ratio. Where missing, the value of ν was assumed to be 0.45.

^c^α: material exponent parameter.

^d^μ: ground shear hyperelastic modulus.

^e^D: compressibility constant.

^f^SCI: spinal cord injury.

^g^Not available.

^h^Values in italics are input based on the identity for isotropic materials, D=3(1-2ν)/(μ{1+ν}), and for linear elastic, μ=E/(2{1+ν}).

### Findings From the FEA Studies of DCM

Of the DCM models, 60% (9/15) specifically focused on ossification of the posterior longitudinal ligament (OPLL), a specific subtype of DCM.

#### Stress and Static Cord Compression

**Figure 3:**
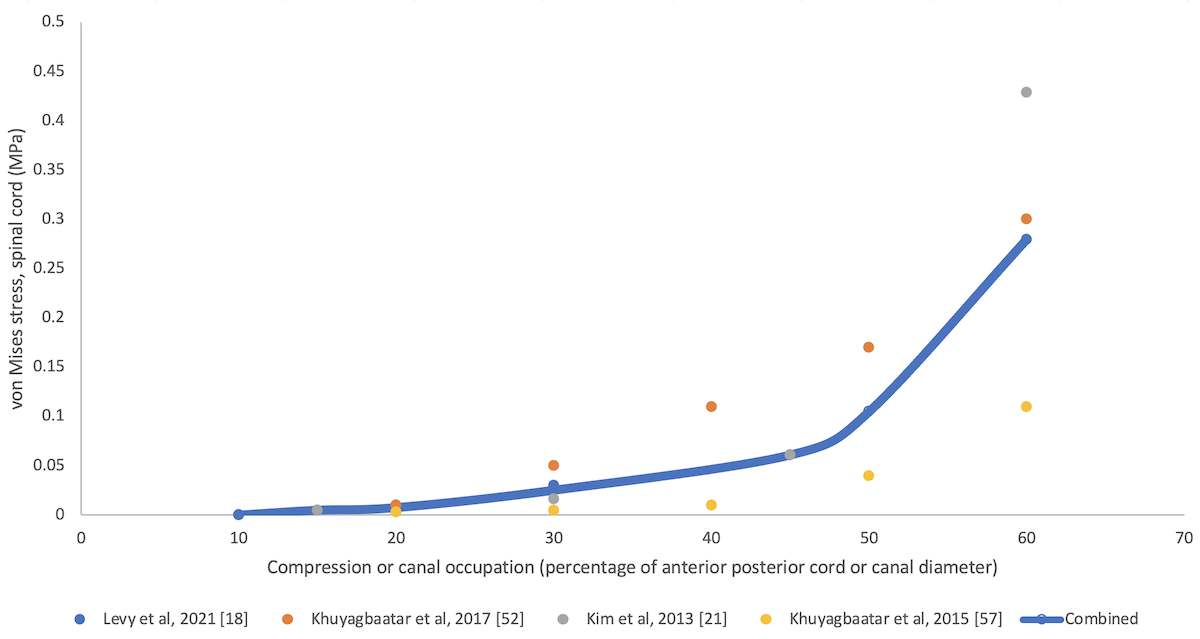
Spinal cord compression and spinal cord stress in degenerative cervical myelopathy models. For models tabulating the von Mises stress at different measures of static compression or canal stenosis (n=4) [18,21,52,57], the values were plotted on a line graph with a line of best fit representing the average value (blue).

A total of 8 models explored the relationship between the amount of static spinal cord compression and spinal cord stress. Kato et al [56] and Kim et al [21] used parametric models of the spinal cord to explore the implications of OPLL (anterior) compression at 2 adjacent vertebrae. The model was constrained posteriorly, reflecting the lamina. They found that the stress increased with increasing cord compression, with an apparent exponential relationship. Minimal stress was detected at <40% but dramatically increased at ≥50%. This relationship was replicated by Nishida et al [91] using posterior compression, by Liang et al [48] simulating a disk prolapse, and in a multisegmental model of OPLL by Khuyagbaatar et al [52,57]. Furthermore, it was replicated in cervical spondylosis by Levy et al [18] (Figure 3 [18,21,52,57]).

**Figure 4:**
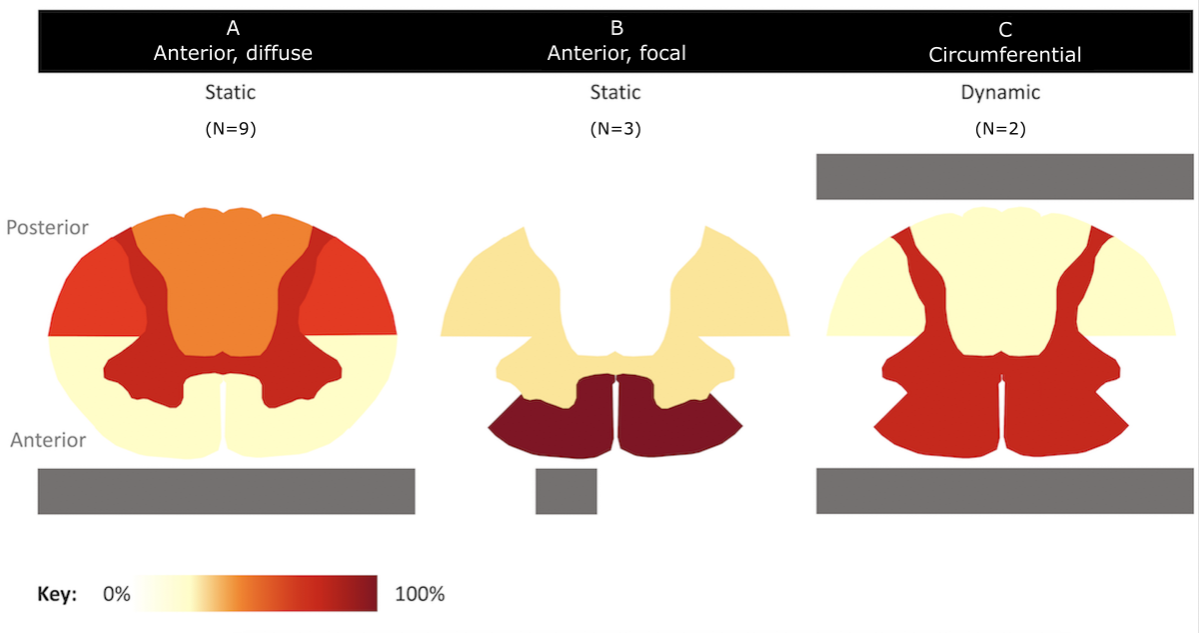
Spinal cord compression and location of spinal cord stress in degenerative cervical myelopathy models. The spinal cord was partitioned, per hemicord, as gray matter and anterior, anterolateral, posterolateral, and posterior white matter. For each study, reporting the cross-sectional distribution of Von Mises stress (n=12) and the location of stress that fell within the top 30% of measured stress was noted. These frequencies were aggregated by compression pattern and displayed for (A) anterior diffuse and static, (B) anterior focal and static, and (C) circumferential and dynamic distribution and location of stress as relative proportions.

Maximal stress was observed in the gray matter and, to a lesser extent, in the lateral and posterior funiculus. Nishida et al [91] observed differences in the stress distribution at low compression rates depending on the spinal cord level related to differing morphology; however, beyond a compression rate of 30%, this was consistent (Figure 4).

Okazaki et al [50] explored the implications of spinal cord aging using a parametric model of the spinal cord. The model was given white and gray matter properties based on a young or aged bovine spinal cord specimen. They observed that stress increased under a low amount of anterior compression in the aged spinal cord and was more widely distributed throughout the gray matter and white matter. In contrast, the gray matter was unaffected in the young specimen.

#### Stress and Dynamic Cord Compression

Nishida et al [22] used a parametric model to explore the implications of ligamentum flavum buckling in neck extension in the context of cervical stenosis. For this, the spinal cord was restricted posteriorly by the ligamentum flavum and then anteriorly, either by a central curvature (representing a disk prolapse) or a flat lateral or flat cross-sectional constraint (representing the ligament). The amount of ligamentum flavum buckling was measured using a kinematic MRI. Spinal cord stress was observed in all scenarios and was maximal using the flat cross-sectional constraint.

Later, Nishida et al [54] used a parametric model of OPLL to demonstrate that while dynamic and static compression alone could stress the spinal cord, they could also act together, although it was unclear whether this was additive or multiplicative. In dynamic compression alone, stress was more restricted to gray matter.

#### Stress and Shape of Cord Compression

Khuyagbaatar et al [57] and Kim et al [21] did not identify any difference in OPLL shape or type with respect to observed spinal cord stress. Furthermore, in the study by Nishida et al [22], the distribution of stress was broadly comparable across the three scenarios affecting the gray matter and anterior and posterolateral aspects of the white matter tracts. In unilateral compression only, the ipsilateral gray matter was affected. Levy et al [18] explored gradually increasing anterior diffuse (broad-based disk), anterior lateral, and circumferential compression using a static multilevel model. Different phenotypes of stress were observed, including peak stress, point of onset, and rate of increase. The highest stress was observed with an anterior diffuse or circumferential compression (Figure 4).

#### Stress and Surgical Decompression

Khuyagbaatar et al [39] used a multisegmental model to explore the implications of hemilaminectomy, laminectomy, and laminoplasty on spinal cord stress following a 1-, 2-, 3-, or 4-level posterior decompression for continuous OPLL. Stress remained elevated following hemilaminectomy but was low and equivalent between laminectomy and laminoplasty. The postoperative deformity was not modeled.

Nishida et al [55] used a parametric model to explore the implications of alignment following posterior decompression for OPLL. They demonstrated that although stress decreased significantly following decompression, it slightly increased in the anterior funiculus, increasing in the gray matter and posterolateral funiculi with progressive deformity. They subsequently replicated this in a separate analysis [51], demonstrating that kyphosis and increased mobility after decompression would elevate the observed stress.

Khuyagbaatar et al [49,52] explored the effects of laminectomy and laminoplasty, respectively, for the treatment of OPLL using a multisegmental static compression model. They demonstrated that all procedures reduced spinal cord stress significantly (>90%), whether in lordotic (K Line positive) or kyphotic deformity (K Line negative) [94]. However, stress was elevated within the exiting C5 nerve root following laminectomy if there was a kyphotic deformity and lateral-type OPLL following laminoplasty. In both instances, the amount of nerve root stress was related to the amount of anterior compression.

Stoner et al [20] used a multisegmental dynamic model (C2-T1) to explore the implications of multilevel C4-7 cervical spondylosis (anterior disk prolapses and osteophyte formation) treated with C4-7 anterior cervical discectomy and fusion (ACDF), laminoplasty, or ACDF with laminectomy. Notably, all procedures caused stress to increase at adjacent levels above those of healthy controls. However, a stand-alone ACDF caused increased stress within the spinal cord at C3 to a level above that of the preoperative DCM model in flexion.

Where possible, these were aggregated, demonstrating that the spinal cord tolerated significant compression before stress increased exponentially (Figure 3 [18,21,52,57]). Aggregating the distributions of stress observed across studies, based on the nature of compression, demonstrated differing stress distributions (Figure 4). For static and diffuse anterior compression, the bilateral posterior white matter and gray matter were the most affected. For static and focal compression, the anterior white matter and, to a lesser extent, the gray matter were most affected. This was observed bilaterally despite a focal or lateral element. For circumferential compression in a dynamic model, the bilateral gray matter and posterior white matter were the most affected.

#### Stress and Tissue Injury

Notably, although differential patterns of stress were observed throughout these DCM models, the levels remained relatively low (<0.5 MPa). DCM FEA models did not explore the relationship between the observed stress and tissue injury.

## Discussion

### Overview

FEA is a promising technique used in DCM, although there remain uncertainties regarding the ideal approach and its clinical interpretation. This review highlights the numerous decisions investigators must make when performing FEA, which can affect findings and underpin the need for a systematic approach, as applied in this study. On the basis of current evidence, we have shortlisted our preferred material property choices for a DCM model and conclude that a distinction between gray and white matter is preferable.

### Principal Findings and Comparison to Prior Work

A total of 15 studies were identified applying FEA to investigate DCM. The insights from these studies broadly align with the current evidence base. First, the spinal cord can tolerate some compression. This is in keeping with clinical practice, where asymptomatic spinal cord compression is far more common [3], and the amount of cord compression is a poor surrogate for disease severity or progression [1]. Second, the movement of the subaxial cervical spine can augment the stress on the spinal cord. This is in keeping with clinical practice, including the concept of dynamic injury and the proposed role of flexion/extension MRI or electrophysiology [95-98]. Finally, it demonstrated the significant effectiveness of decompression surgery, regardless of the technique, and the comparatively minor gains of using one technique over the other. This is in keeping with clinical practice, where high-quality comparisons of anterior versus posterior surgery are equivalent, and currently, there is no strong evidence that routine stabilization (eg, instrumented fusion vs laminoplasty vs laminectomy or ACDF vs ACDF with a plate) is required [99-102], all pointing toward the need for a personalized surgical approach [15].

Furthermore, although more nuanced findings were proposed by the identified FEA studies and this would require in vivo corroboration, the application of FEA in DCM appears well founded overall. More widely, it also seems potentially valuable and timely. The pathobiology of DCM is poorly understood, with its investigation being among the top 10 global research priorities [1]. Current preclinical models have many limitations. For example, common recent models use an expandable polymer inserted behind the spinal cord and within the canal to cause cervical myelopathy. Therefore, this does not model anterior compression, nor does it truly represent a chronic injury mechanism. Furthermore, in clinical practice, clinical decisions are based on imperfect tools [103]. For example, structural MRI in a supine position defines the nature of degenerative changes but not if, where, or how an SCI occurs. FEA could change this, particularly given the parallel advances in the automatic segmentation of MRI [12].

Furthermore, while this review highlights that FEA is a versatile technique, investigators must make many decisions regarding how it is applied. These decisions can alter the findings and, therefore, must be carefully considered. At this stage, there seem to be only a few pervasive insights. First, it seems prudent to model the white matter and gray matter separately. Ichihara et al [73] demonstrated that these structures have differing material properties, and how they are defined alters the observed stress and strain. Furthermore, these structures age differently, as shown by Ozawa et al [76]. Histological studies of DCM have shown differing disease features among the white matter and gray matter, with the gray matter being the focus of more significant cellular changes [9]. Moreover, aging is an important factor in DCM, associated with greater disease severity, a greater rate of progression, and poorer response to treatment [104]. There are also early indicators that accelerating aging is a pathological process [1]. Therefore, the observation that the gray matter was unaffected in the younger spinal cord specimen is noteworthy [34,45,50].

Second, while some models have chosen to use linear elastic equations, time-independent hyperelastic models more closely reflected the known material properties of the spinal cord. These, or simply tabulated data, were generally adopted by DCM studies and supported by a single study that evaluated different approaches [38]. Conceptually, taking a more faithful approach to modeling the spinal cord material properties is likely to be more applicable to DCM and its etiology, as contrasted with traumatic SCI, spinal cord stress may be below the limits for tissue injury (eg, asymptomatic spinal cord compression), and above (eg, DCM). It is worth noting that none of these approaches considers the impact of repetitive injuries, and it is likely that time dependence in modeling is relevant [1]. Given the timeline of DCM pathogenesis (years), this is likely beyond the normal material scales.

Finally, similar to DCM, as the stresses involved are well below the elastic limit of the bone, the vertebrae can be modeled simply as rigid bodies. The critical aspect for bones is instead the way that their geometry and movement affect the loading on the soft tissues.

However, there remain many uncertainties for further evaluation. These include the role of spinal cord oscillation, the appropriateness of the reference material properties for DCM, and the relationship between the measured stress and tissue injury. First, no studies specifically consider spinal cord oscillations [105]. The spinal cord oscillates cranio-caudally with heart rate. Recent imaging studies have indicated that this increased in the context of symptomatic stenosis, the nature of which may correlate with clinical measures of disease severity [106,107]. Spinal cord oscillation would likely result in a shear force on the spinal cord.

Second, it is uncertain how applicable the material properties are to DCM. Most elements are based on young healthy tissue references. In contrast, the ligaments and disks, for example, in DCM, are often degenerated and calcified, and, as aforementioned, the structure of the spinal cord is also recognized to change with age.

However, most importantly, none of these studies have specifically explored how the measured stress is related to tissue injury. Bridging this gap is critical, not only to fully confirm the appropriateness of FEA for DCM but also to guide its clinical interpretation [108]. All biological systems will have some baseline stress or strain; therefore, establishing disease thresholds will be critical to its development. The parallel development of in vivo techniques to measure tissue injury can complement this, for example, microstructural MRI and the less developed but promising serum and CSF biomarkers; however, this requires further prospective study.

### Limitations

This study has some limitations. First, the search strategy focused on FEA models of the spinal cord and used citations to identify the source articles for all anatomical elements. Consequently, relevant source articles on the behavior of anatomical elements may have been missed. This is more likely for elements that were further removed from the spinal cord, such as the IVD, and experiments published more recently. This was a pragmatic decision based on the fact that existing investigators would likely have the best perspective on the literature, that this is a small research field, and that detailed biomechanical data on elements such as the IVD were unlikely to be so relevant. Consistent decisions across different research groups and findings across source articles would endorse this. Furthermore, due to the nature of our synthesis, we were unable to update our search. Although this may result in the omission of newer FEA articles, we believe that our review provides a useful approach for future investigators aiming to use FEA in DCM. Second, the methods used to shortlist source articles represent a framework we developed for the purpose of building a DCM FEA model. Again, the popularity of the shortlisted articles across research groups provides some external validation, but it is possible that different investigators would reach different conclusions. For this reason, all source articles are listed in Multimedia Appendix 6, with their respective direct object identifiers. Third, this review aggregates data from a range of different experimental approaches and aims. Therefore, the analysis is largely qualitative, adhering to the SWiM guidelines [26]. Consequently, some conclusions, such as the relationship between the nature of spinal cord compression and stress distribution, remain tentative.

### Conclusions

FEA has significant potential to help unlock uncertainties around the pathophysiology of DCM and inform clinical care. Currently, the application of FEA to DCM remains in its infancy. This review has adopted an intensive and iterative approach to help future investigators use FEA in DCM, including the aggregation of experimental data reporting on material properties and how they have been interpreted thus far. While single recommendations have been made, they have their limitations. The choice of material properties will influence the model performance, and investigators should consider their decisions carefully, particularly as new evidence emerges. More broadly, the methodology used in this review may be relevant to future updates and other clinical FEA initiatives when selecting material properties.

## Appendix

Multimedia Appendix 1 PRISMA-ScR (Preferred Reporting Items for Systematic Reviews and Meta-Analyses extension for Scoping Reviews) checklist.

Multimedia Appendix 2 Search strategy.

Multimedia Appendix 3 The quality assessment tool developed by the authors.

Multimedia Appendix 4 Comparison of modeling decisions.

Multimedia Appendix 5 Comparison of chosen equation and reference material property study.

Multimedia Appendix 6 Material properties of other anatomical elements.

Multimedia Appendix 7 Interactive network files.

Multimedia Appendix 8 Higher-resolution version of Figure 2.

## Abbreviations

ACDF: anterior cervical discectomy and fusion
CSF: cerebrospinal fluid
DCM: degenerative cervical myelopathy
FEA: finite element analysis
IVD: intervertebral disk
MRI: magnetic resonance imaging
OPLL: ossification of the posterior longitudinal ligament
PRISMA-ScR: Preferred Reporting Items for Systematic Reviews and Meta-Analyses extension for Scoping Reviews
SCI: spinal cord injury
SWiM: Synthesis Without Meta-Analysis

## Data Availability

The data sets generated during and analyzed during this study are available from the corresponding author on reasonable request.

## References

[1] Davies BM, Mowforth O, Gharooni AA, Tetreault L, Nouri A, Dhillon RS, Bednarik J, Martin AR, Young A, Takahashi H, Boerger TF, Newcombe VF, Zipser CM, Freund P, Koljonen PA, Rodrigues-Pinto R, Rahimi-Movaghar V, Wilson JR, Kurpad SN, Fehlings MG, Kwon BK, Harrop JS, Guest JD, Curt A, Kotter MR (2022). A new framework for investigating the biological basis of degenerative cervical myelopathy [AO spine RECODE-DCM research priority number 5]: mechanical stress, vulnerability and time. Global Spine J.

[2] Davies BM, Munro C, Khan DZ, Fitzpatrick SM, Hilton B, Mowforth OD, McNair AG, Sadler I, Kotter MR (2022). Outcomes of degenerative cervical myelopathy from the perspective of persons living with the condition: findings of a semistructured interview process with partnered internet survey. Global Spine J.

[3] Smith SS, Stewart ME, Davies BM, Kotter MR (2021). The prevalence of asymptomatic and symptomatic spinal cord compression on magnetic resonance imaging: a systematic review and meta-analysis. Global Spine J.

[4] Davies BM, Mowforth OD, Smith EK, Kotter MR (2018). Degenerative cervical myelopathy. BMJ.

[5] Fehlings MG, Tetreault LA, Riew KD, Middleton JW, Aarabi B, Arnold PM, Brodke DS, Burns AS, Carette S, Chen R, Chiba K, Dettori JR, Furlan JC, Harrop JS, Holly LT, Kalsi-Ryan S, Kotter M, Kwon BK, Martin AR, Milligan J, Nakashima H, Nagoshi N, Rhee J, Singh A, Skelly AC, Sodhi S, Wilson JR, Yee A, Wang JC (2017). A clinical practice guideline for the management of patients with degenerative cervical myelopathy: recommendations for patients with mild, moderate, and severe disease and nonmyelopathic patients with evidence of cord compression. Global Spine J.

[6] Fehlings MG, Ibrahim A, Tetreault L, Albanese V, Alvarado M, Arnold P, Barbagallo G, Bartels R, Bolger C, Defino H, Kale S, Massicotte E, Moraes O, Scerrati M, Tan G, Tanaka M, Toyone T, Yukawa Y, Zhou Q, Zileli M, Kopjar B (2015). A global perspective on the outcomes of surgical decompression in patients with cervical spondylotic myelopathy. Spine.

[7] Oh T, Lafage R, Lafage V, Protopsaltis T, Challier V, Shaffrey C, Kim HJ, Arnold P, Chapman J, Schwab F, Massicotte E, Yoon T, Bess S, Fehlings M, Smith J, Ames C (2017). Comparing quality of life in cervical spondylotic myelopathy with other chronic debilitating diseases using the short form survey 36-health survey. World Neurosurg.

[8] Davies BM, Phillips R, Clarke D, Furlan JC, Demetriades AK, Milligan J, Witiw CD, Harrop JS, Aarabi B, Kurpad SN, Guest JD, Wilson JR, Kwon BK, Vaccaro AR, Fehlings MG, Rahimi-Movaghar V, Kotter MR (2022). Establishing the socio-economic impact of degenerative cervical myelopathy is fundamental to improving outcomes [AO spine RECODE-DCM research priority number 8]. Global Spine J.

[9] Badhiwala JH, Ahuja CS, Akbar MA, Witiw CD, Nassiri F, Furlan JC, Curt A, Wilson JR, Fehlings MG (2020). Degenerative cervical myelopathy - update and future directions. Nat Rev Neurol.

[10] Martin AR, De Leener B, Cohen-Adad J, Cadotte DW, Nouri A, Wilson JR, Tetreault L, Crawley AP, Mikulis DJ, Ginsberg H, Fehlings MG (2018). Can microstructural MRI detect subclinical tissue injury in subjects with asymptomatic cervical spinal cord compression? A prospective cohort study. BMJ Open.

[11] Tetreault L, Kopjar B, Côté P, Arnold P, Fehlings MG (2015). A clinical prediction rule for functional outcomes in patients undergoing surgery for degenerative cervical myelopathy: analysis of an international prospective multicenter data set of 757 subjects. J Bone Joint Surg Am.

[12] Martin AR, Tetreault L, Nouri A, Curt A, Freund P, Rahimi-Movaghar V, Wilson JR, Fehlings MG, Kwon BK, Harrop JS, Davies BM, Kotter MR, Guest JD, Aarabi B, Kurpad SN (2022). Imaging and electrophysiology for degenerative cervical myelopathy [AO spine RECODE-DCM research priority number 9]. Global Spine J.

[13] Henderson FC, Geddes JF, Vaccaro AR, Woodard E, Berry KJ, Benzel EC (2005). Stretch-associated injury in cervical spondylotic myelopathy: new concept and review. Neurosurgery.

[14] Nouri A, Martin AR, Tetreault L, Nater A, Kato S, Nakashima H, Nagoshi N, Reihani-Kermani H, Fehlings MG (2017). MRI analysis of the combined prospectively collected AOSpine North America and international data: the prevalence and spectrum of pathologies in a global cohort of patients with degenerative cervical myelopathy. Spine (Phila Pa 1976).

[15] Rodrigues-Pinto R, Montenegro TS, Davies BM, Kato S, Kawaguchi Y, Ito M, Zileli M, Kwon BK, Fehlings MG, Koljonen PA, Kurpad SN, Guest JD, Aarabi B, Rahimi-Movaghar V, Wilson JR, Kotter MR, Harrop JS (2022). Optimizing the application of surgery for degenerative cervical myelopathy [AO spine RECODE-DCM research priority number 10]. Global Spine J.

[16] Jones CF, Clarke EC (2019). Engineering approaches to understanding mechanisms of spinal column injury leading to spinal cord injury. Clin Biomech (Bristol, Avon).

[17] Jones AC, Wilcox RK (2008). Finite element analysis of the spine: towards a framework of verification, validation and sensitivity analysis. Med Eng Phys.

[18] Lévy S, Baucher G, Roche PH, Evin M, Callot V, Arnoux PJ (2021). Biomechanical comparison of spinal cord compression types occurring in Degenerative Cervical Myelopathy. Clin Biomech (Bristol, Avon).

[19] Munn Z, Peters MD, Stern C, Tufanaru C, McArthur A, Aromataris E (2018). Systematic review or scoping review? Guidance for authors when choosing between a systematic or scoping review approach. BMC Med Res Methodol.

[20] Stoner KE, Abode-Iyamah KO, Fredericks DC, Viljoen S, Howard MA, Grosland NM (2020). A comprehensive finite element model of surgical treatment for cervical myelopathy. Clin Biomech (Bristol, Avon).

[21] Kim YH, Khuyagbaatar B, Kim K (2013). Biomechanical effects of spinal cord compression due to ossification of posterior longitudinal ligament and ligamentum flavum: a finite element analysis. Med Eng Phys.

[22] Nishida N, Kato Y, Imajo Y, Kawano S, Taguchi T (2013). Biomechanical analysis of cervical spondylotic myelopathy: the influence of dynamic factors and morphometry of the spinal cord. J Spinal Cord Med.

[23] Li Z, Liu H, Yang M, Zhang W (2021). A biomechanical analysis of four anterior cervical techniques to treating multilevel cervical spondylotic myelopathy: a finite element study. BMC Musculoskelet Disord.

[24] Sunstein CR, Kahneman D, Sibony O (2021). Noise: A Flaw in Human Judgment.

[25] Stang A (2010). Critical evaluation of the Newcastle-Ottawa scale for the assessment of the quality of nonrandomized studies in meta-analyses. Eur J Epidemiol.

[26] Campbell M, McKenzie JE, Sowden A, Katikireddi SV, Brennan SE, Ellis S, Hartmann-Boyce J, Ryan R, Shepperd S, Thomas J, Welch V, Thomson H (2020). Synthesis without meta-analysis (SWiM) in systematic reviews: reporting guideline. BMJ.

[27] Yu QQ, Liu SQ, Wang JJ, Xu ML, Zhang WX, Cheng LM, Zhu R (2021). Effects of a contusion load on spinal cord with different curvatures. Comput Methods Biomech Biomed Engin.

[28] Beauséjour MH, Wagnac E, Arnoux PJ, Thiong JM, Petit Y (2022). Numerical investigation of spinal cord injury after flexion-distraction injuries at the cervical spine. J Biomech Eng.

[29] Jannesar S, Salegio EA, Beattie MS, Bresnahan JC, Sparrey CJ (2021). Correlating tissue mechanics and spinal cord injury: patient-specific finite element models of unilateral cervical contusion spinal cord injury in non-human primates. J Neurotrauma.

[30] Zhu R, Chen YH, Yu QQ, Liu SQ, Wang JJ, Zeng ZL, Cheng LM (2020). Effects of contusion load on cervical spinal cord: a finite element study. Math Biosci Eng.

[31] Arhiptsov K, Marom G (2021). Numerical models of spinal cord trauma: the effect of cerebrospinal fluid pressure and epidural fat on the results. J Neurotrauma.

[32] Bailly N, Diotalevi L, Beauséjour MH, Wagnac É, Mac-Thiong JM, Petit Y (2020). Numerical investigation of the relative effect of disc bulging and ligamentum flavum hypertrophy on the mechanism of central cord syndrome. Clin Biomech (Bristol, Avon).

[33] Sparrey CJ, Salegio EA, Camisa W, Tam H, Beattie MS, Bresnahan JC (2016). Mechanical design and analysis of a unilateral cervical spinal cord contusion injury model in non-human primates. J Neurotrauma.

[34] Sparrey CJ, Manley GT, Keaveny TM (2009). Effects of white, grey, and pia mater properties on tissue level stresses and strains in the compressed spinal cord. J Neurotrauma.

[35] Lam CJ, Assinck P, Liu J, Tetzlaff W, Oxland TR (2014). Impact depth and the interaction with impact speed affect the severity of contusion spinal cord injury in rats. J Neurotrauma.

[36] Yan YB, Qi W, Wu ZX, Qiu TX, Teo EC, Lei W (2012). Finite element study of the mechanical response in spinal cord during the thoracolumbar burst fracture. PLoS One.

[37] Li XF, Dai LY (2010). Acute central cord syndrome: injury mechanisms and stress features. Spine (Phila Pa 1976).

[38] Jannesar S, Nadler B, Sparrey CJ (2016). The transverse isotropy of spinal cord white matter under dynamic load. J Biomech Eng.

[39] Khuyagbaatar B, Kim K, Man Park W, Hyuk Kim Y (2016). Biomechanical behaviors in three types of spinal cord injury mechanisms. J Biomech Eng.

[40] Khuyagbaatar B, Kim K, Hyuk Kim Y (2014). Effect of bone fragment impact velocity on biomechanical parameters related to spinal cord injury: a finite element study. J Biomech.

[41] Greaves CY, Gadala MS, Oxland TR (2008). A three-dimensional finite element model of the cervical spine with spinal cord: an investigation of three injury mechanisms. Ann Biomed Eng.

[42] Czyz M, Scigala K, Jarmundowicz W, Beidziński R (2008). The biomechanical analysis of the traumatic cervical spinal cord injury using finite element approach. Acta Bioeng Biomech.

[43] Maikos JT, Qian Z, Metaxas D, Shreiber DI (2008). Finite element analysis of spinal cord injury in the rat. J Neurotrauma.

[44] Scifert J, Totoribe K, Goel V, Huntzinger J (2002). Spinal cord mechanics during flexion and extension of the cervical spine: a finite element study. Pain Phys.

[45] Fournely M, Petit Y, Wagnac E, Evin M, Arnoux PJ (2020). Effect of experimental, morphological and mechanical factors on the murine spinal cord subjected to transverse contusion: a finite element study. PLoS One.

[46] Persson C, Summers J, Hall RM (2011). The importance of fluid-structure interaction in spinal trauma models. J Neurotrauma.

[47] Wilcox RK, Allen DJ, Hall RM, Limb D, Barton DC, Dickson RA (2004). A dynamic investigation of the burst fracture process using a combined experimental and finite element approach. Eur Spine J.

[48] Liang D, Tu GJ, Han YX, Guo DW (2021). Accurate simulation of the herniated cervical intervertebral disc using controllable expansion: a finite element study. Comput Methods Biomech Biomed Eng.

[49] Khuyagbaatar B, Kim K, Purevsuren T, Lee SH, Kim YH (2018). Biomechanical effects on cervical spinal cord and nerve root following laminoplasty for ossification of the posterior longitudinal ligament in the cervical spine: a comparison between open-door and double-door laminoplasty using finite element analysis. J Biomech Eng.

[50] Okazaki T, Kanchiku T, Nishida N, Ichihara K, Sakuramoto I, Ohgi J, Funaba M, Imajo Y, Suzuki H, Chen X, Taguchi T (2018). Age-related changes of the spinal cord: a biomechanical study. Exp Ther Med.

[51] Nishida N, Kanchiku T, Kato Y, Imajo Y, Suzuki H, Yoshida Y, Ohgi J, Chen X, Taguchi T (2017). Cervical ossification of the posterior longitudinal ligament: factors affecting the effect of posterior decompression. J Spinal Cord Med.

[52] Khuyagbaatar B, Kim K, Park WM, Kim YH (2017). Biomechanical investigation of post-operative C5 palsy due to ossification of the posterior longitudinal ligament in different types of cervical spinal alignment. J Biomech.

[53] Khuyagbaatar B, Kim K, Park WM, Kim YH (2016). Effect of posterior decompression extent on biomechanical parameters of the spinal cord in cervical ossification of the posterior longitudinal ligament. Proc Inst Mech Eng H.

[54] Nishida N, Kanchiku T, Kato Y, Imajo Y, Yoshida Y, Kawano S, Taguchi T (2015). Cervical ossification of the posterior longitudinal ligament: biomechanical analysis of the influence of static and dynamic factors. J Spinal Cord Med.

[55] Nishida N, Kanchiku T, Kato Y, Imajo Y, Yoshida Y, Kawano S, Taguchi T (2014). Biomechanical analysis of cervical myelopathy due to ossification of the posterior longitudinal ligament: effects of posterior decompression and kyphosis following decompression. Exp Ther Med.

[56] Kato Y, Kanchiku T, Imajo Y, Kimura K, Ichihara K, Kawano S, Hamanaka D, Yaji K, Taguchi T (2010). Biomechanical study of the effect of degree of static compression of the spinal cord in ossification of the posterior longitudinal ligament. J Neurosurg Spine.

[57] Khuyagbaatar B, Kim K, Park WM, Kim YH (2015). Influence of sagittal and axial types of ossification of posterior longitudinal ligament on mechanical stress in cervical spinal cord: a finite element analysis. Clin Biomech (Bristol, Avon).

[58] Henao J, Labelle H, Arnoux PJ, Aubin CE (2018). Biomechanical simulation of stresses and strains exerted on the spinal cord and nerves during scoliosis correction maneuvers. Spine Deform.

[59] Henao J, Aubin CE, Labelle H, Arnoux PJ (2016). Patient-specific finite element model of the spine and spinal cord to assess the neurological impact of scoliosis correction: preliminary application on two cases with and without intraoperative neurological complications. Comput Methods Biomech Biomed Eng.

[60] Bertram CD (2010). Evaluation by fluid/structure-interaction spinal-cord simulation of the effects of subarachnoid-space stenosis on an adjacent syrinx. J Biomech Eng.

[61] Bertram CD, Bilston LE, Stoodley MA (2008). Tensile radial stress in the spinal cord related to arachnoiditis or tethering: a numerical model. Med Biol Eng Comput.

[62] Kato Y, Kataoka H, Ichihara K, Imajo Y, Kojima T, Kawano S, Hamanaka D, Yaji K, Taguchi T (2008). Biomechanical study of cervical flexion myelopathy using a three-dimensional finite element method. J Neurosurg Spine.

[63] Kato Y, Kanchiku T, Imajo Y, Ichinara K, Kawano S, Hamanama D, Yaji K, Taguchi T (2009). Flexion model simulating spinal cord injury without radiographic abnormality in patients with ossification of the longitudinal ligament: the influence of flexion speed on the cervical spine. J Spinal Cord Med.

[64] Mitsuhashi N, Fujieda K, Tamura T, Kawamoto S, Takagi T, Okubo K (2009). BodyParts3D: 3D structure database for anatomical concepts. Nucleic Acids Res.

[65] Frostell A, Hakim R, Thelin EP, Mattsson P, Svensson M (2016). A review of the segmental diameter of the healthy human spinal cord. Front Neurol.

[66] Barker JB, Cronin DS, Chandrashekar N (2014). High rotation rate behavior of cervical spine segments in flexion and extension. J Biomech Eng.

[67] Onan OA, Heggeness MH, Hipp JA (1998). A motion analysis of the cervical facet joint. Spine (Phila Pa 1976).

[68] Grauer JN, Panjabi MM, Cholewicki J, Nibu K, Dvorak J (1997). Whiplash produces an S-shaped curvature of the neck with hyperextension at lower levels. Spine (Phila Pa 1976).

[69] Moroney SP, Schultz AB, Miller JA, Andersson GB (1988). Load-displacement properties of lower cervical spine motion segments. J Biomech.

[70] Stoner KE, Abode-Iyamah KO, Magnotta VA, Howard MA, Grosland NM (2019). Measurement of in vivo spinal cord displacement and strain fields of healthy and myelopathic cervical spinal cord. J Neurosurg Spine.

[71] Ozawa H, Matsumoto T, Ohashi T, Sato M, Kokubun S (2004). Mechanical properties and function of the spinal pia mater. J Neurosurg Spine.

[72] Ichihara K, Taguchi T, Sakuramoto I, Kawano S, Kawai S (2003). Mechanism of the spinal cord injury and the cervical spondylotic myelopathy: new approach based on the mechanical features of the spinal cord white and gray matter. J Neurosurg.

[73] Ichihara K, Taguchi T, Shimada Y, Sakuramoto I, Kawano S, Kawai S (2001). Gray matter of the bovine cervical spinal cord is mechanically more rigid and fragile than the white matter. J Neurotrauma.

[74] Bilston LE, Thibault LE (1995). The mechanical properties of the human cervical spinal cordIn Vitro. Ann Biomed Eng.

[75] Tunturi AR (1978). Elasticity of the spinal cord, pia, and denticulate ligament in the dog. J Neurosurg.

[76] Ozawa H, Matsumoto T, Ohashi T, Sato M, Kokubun S (2001). Comparison of spinal cord gray matter and white matter softness: measurement by pipette aspiration method. J Neurosurg.

[77] Kimpara H, Nakahira Y, Iwamoto M, Miki K, Ichihara K, Kawano SI, Taguchi T (2006). Investigation of anteroposterior head-neck responses during severe frontal impacts using a brain-spinal cord complex FE model. Stapp Car Crash J.

[78] Hong JY, Suh SW, Park SY, Modi HN, Rhyu IJ, Kwon S, Yu H, Byun J (2011). Analysis of dural sac thickness in human spine-cadaver study with confocal infrared laser microscope. Spine J.

[79] Zarzur E (1996). Mechanical properties of the human lumbar dura mater. Arq Neuropsiquiatr.

[80] Persson C, Evans S, Marsh R, Summers JL, Hall RM (2010). Poisson's ratio and strain rate dependency of the constitutive behavior of spinal dura mater. Ann Biomed Eng.

[81] Wilcox RK, Bilston LE, Barton DC, Hall RM (2003). Mathematical model for the viscoelastic properties of dura mater. J Orthop Sci.

[82] Tubbs RS, Salter G, Grabb PA, Oakes WJ (2001). The denticulate ligament: anatomy and functional significance. J Neurosurg.

[83] Bloomfield I, Johnston IH, Bilston LE (1998). Effects of proteins, blood cells and glucose on the viscosity of cerebrospinal fluid. Pediatr Neurosurg.

[84] Brydon HL, Hayward R, Harkness W, Bayston R (1995). Physical properties of cerebrospinal fluid of relevance to shunt function. 1: the effect of protein upon CSF viscosity. Br J Neurosurg.

[85] Przybylski GJ, Carlin GJ, Patel PR, Woo SL (1996). Human anterior and posterior cervical longitudinal ligaments possess similar tensile properties. J Orthop Res.

[86] Yoganandan N, Kumaresan S, Pintar FA (2000). Geometric and mechanical properties of human cervical spine ligaments. J Biomech Eng.

[87] Yoganandan N, Pintar F, Butler J, Reinartz J, Sances A Jr, Larson SJ (1989). Dynamic response of human cervical spine ligaments. Spine (Phila Pa 1976).

[88] Kulkarni VA, Massie JB, Zauner F, Murphy M, Akeson WH (2007). Novel biomechanical quantification methodology for lumbar intraforaminal spinal nerve adhesion in a laminectomy and disc injury rat model. J Neurosci Methods.

[89] Singh A, Lu Y, Chen C, Cavanaugh JM (2006). Mechanical properties of spinal nerve roots subjected to tension at different strain rates. J Biomech.

[90] Spilker RL, Jakobs DM, Schultz AB (1986). Material constants for a finite element model of the intervertebral disk with a fiber composite annulus. J Biomech Eng.

[91] Nishida N, Kanchiku T, Imajo Y, Suzuki H, Yoshida Y, Kato Y, Nakashima D, Taguchi T (2016). Stress analysis of the cervical spinal cord: impact of the morphology of spinal cord segments on stress. J Spinal Cord Med.

[92] Persson C, Evans S, Marsh R, Summers JL, Hall RM (2010). Poisson's ratio and strain rate dependency of the constitutive behavior of spinal dura mater. Ann Biomed Eng.

[93] Jones CF, Kroeker SG, Cripton PA, Hall RM (2008). The effect of cerebrospinal fluid on the biomechanics of spinal cord: an ex vivo bovine model using bovine and physical surrogate spinal cord. Spine.

[94] Taniyama T, Hirai T, Yamada T, Yuasa M, Enomoto M, Yoshii T, Kato T, Kawabata S, Inose H, Okawa A (2013). Modified K-line in magnetic resonance imaging predicts insufficient decompression of cervical laminoplasty. Spine.

[95] Nouri A, Tetreault L, Singh A, Karadimas SK, Fehlings MG (2015). Degenerative cervical myelopathy: epidemiology, genetics, and pathogenesis. Spine.

[96] Gondar R, Nouri A, Jannelli G, Schaller K, Tessitore E (2021). Does spondylolisthesis affect severity and outcome of degenerative cervical myelopathy? A systematic review and meta-analysis. Global Spine J.

[97] Park D, Kim BH, Cho J, Yang JW, Yang DH, Kim MS, Kwon HD, Lee SE (2021). Diagnostic role of flexion-extension central motor conduction time in cervical spondylotic myelopathy. Spine (Phila Pa 1976).

[98] Kolcun JP, Chieng LO, Madhavan K, Wang MY (2017). The role of dynamic magnetic resonance imaging in cervical spondylotic myelopathy. Asian Spine J.

[99] Ghogawala Z, Terrin N, Dunbar MR, Breeze JL, Freund KM, Kanter AS, Mummaneni PV, Bisson EF, Barker FG 2nd, Schwartz JS, Harrop JS, Magge SN, Heary RF, Fehlings MG, Albert TJ, Arnold PM, Riew KD, Steinmetz MP, Wang MC, Whitmore RG, Heller JG, Benzel EC (2021). Effect of ventral vs dorsal spinal surgery on patient-reported physical functioning in patients with cervical spondylotic myelopathy: a randomized clinical trial. JAMA.

[100] Wang J, Wo J, Wen J, Zhang L, Xu W, Wang X (2022). Laminoplasty versus laminectomy with fusion for treatment of multilevel cervical compressive myelopathy: an updated meta-analysis. Postgrad Med J.

[101] Lao L, Zhong G, Li X, Qian L, Liu Z (2013). Laminoplasty versus laminectomy for multi-level cervical spondylotic myelopathy: a systematic review of the literature. J Orthop Surg Res.

[102] Cheung ZB, Gidumal S, White S, Shin J, Phan K, Osman N, Bronheim R, Vargas L, Kim JS, Cho SK (2019). Comparison of anterior cervical discectomy and fusion with a stand-alone interbody cage versus a conventional cage-plate technique: a systematic review and meta-analysis. Global Spine J.

[103] Akter F, Yu X, Qin X, Yao S, Nikrouz P, Syed YA, Kotter M (2020). The pathophysiology of degenerative cervical myelopathy and the physiology of recovery following decompression. Front Neurosci.

[104] Grodzinski B, Durham R, Mowforth O, Stubbs D, Kotter MR, Davies BM (2021). The effect of ageing on presentation, management and outcomes in degenerative cervical myelopathy: a systematic review. Age Ageing.

[105] Mikulis DJ, Wood ML, Zerdoner OA, Poncelet BP (1994). Oscillatory motion of the normal cervical spinal cord. Radiology.

[106] Hupp M, Pfender N, Vallotton K, Rosner J, Friedl S, Zipser CM, Sutter R, Klarhöfer M, Spirig JM, Betz M, Schubert M, Freund P, Farshad M, Curt A (2021). The restless spinal cord in degenerative cervical myelopathy. Am J Neuroradiol.

[107] Vavasour IM, Meyers SM, MacMillan EL, Mädler B, Li DK, Rauscher A, Vertinsky T, Venu V, MacKay AL, Curt A (2014). Increased spinal cord movements in cervical spondylotic myelopathy. Spine J.

[108] Kolias AG, Honeybul S (2021). Traumatic Brain Injury: Science, Practice, Evidence and Ethics.

