# Supplementary material for: Finite Element Analysis for Degenerative Cervical Myelopathy: Scoping Review of the Current Findings and Design Approaches, Including Recommendations on the Choice of Material Properties
Source: JMIR Biomed Eng. 2024 Mar 28;9:e48146. doi: 10.2196/48146 (PMC11041437; doi:10.2196/48146)
Supplement: Multimedia Appendix 2 [file biomedeng_v9i1e48146_app2.docx]

**Multimedia Appendix 2: Search Strategy:**

(‘finite element analysis’ or ‘biomechanical analysis’) AND (‘spinal cord’)

MEDLINE (OvidSP, 1946 to 1^st^ September 2021)

EMBASE (OvidSP, 1974 to 1^st^ September 2021)
