# Supplementary material for: Finite Element Analysis for Degenerative Cervical Myelopathy: Scoping Review of the Current Findings and Design Approaches, Including Recommendations on the Choice of Material Properties
Source: JMIR Biomed Eng. 2024 Mar 28;9:e48146. doi: 10.2196/48146 (PMC11041437; doi:10.2196/48146)
Supplement: Multimedia Appendix 3 [file biomedeng_v9i1e48146_app3.docx]

**Multimedia Appendix 3: Author Developed Quality Assessment Tool**

Author developed, framework for appraising the quality of source articles for the purpose of developing a human DCM FEA mode.

| **Factor** | **Ranked Categories**, from first to last preference | | |
| --- | --- | --- | --- |
| Investigation Method | Tissue | Imaging |  |
| Study Participant | Human | Large Animal (E.g. Cow, Sheep or Pig) | Small Animal (E.g. Rodent) |
| Aged to >2/3s life expectancy | Yes | No | ? |
| Spine Region | Cervical | Thoracic | Other |
| Range of Material Laws Explored | Yes | No | ? |
| Explored with Finite Element Model | Yes | No | ? |
| Risk of Selection Bias | Yes | No | ? |
| Risk of Reporting Bias | Yes | No | ? |
| Other Risk of Concern | Yes | No | ? |
