# Supplementary material for: Finite Element Analysis for Degenerative Cervical Myelopathy: Scoping Review of the Current Findings and Design Approaches, Including Recommendations on the Choice of Material Properties
Source: JMIR Biomed Eng. 2024 Mar 28;9:e48146. doi: 10.2196/48146 (PMC11041437; doi:10.2196/48146)
Supplement: Multimedia Appendix 4 [file biomedeng_v9i1e48146_app4.docx]

Multimedia Appendix 4: Comparison of Modelling Decisions

4.1 Bar chart of the anatomical components contained within identified models overall. Data is presented as a proportion of overall studies (N=42). IVD: Intervertebral Disc. CSF: Cerebrospinal Fluid. LF: Ligamentum Flavum. PLL: Posterior Longitudinal Ligament.

4.2 Clustered bar chart of the anatomical components contained within identified models, by disease. Data is presented as a proportion of each disease group; SCI (23, 55%), DCM (15, 36%) and other. Other represents the Scoliosis, Syringomyelia and Flexion Myelopathy models combined. IVD: Intervertebral Disc. CSF: Cerebrospinal Fluid. LF: Ligamentum Flavum. PLL: Posterior Longitudinal Ligament.

4.3 Line chart, with 5 year moving average of the number of FEA models published with a specific anatomical component. The solid black line represents the overall number of FEA model published that year. The choice of anatomical elements has not changed over time. IVD: Intervertebral Disc. CSF: Cerebrospinal Fluid. LF: Ligamentum Flavum. PLL: Posterior Longitudinal Ligament.

4.4 Bar chart of the proportion of models that incorporated physiological movement of the spinal column. Data is presented as a proportion of each disease group; SCI (23, 55%), DCM (15, 36%) and other. Other represents the Scoliosis, Syringomyelia and Flexion Myelopathy models combined.
