# Supplementary material for: Finite Element Analysis for Degenerative Cervical Myelopathy: Scoping Review of the Current Findings and Design Approaches, Including Recommendations on the Choice of Material Properties
Source: JMIR Biomed Eng. 2024 Mar 28;9:e48146. doi: 10.2196/48146 (PMC11041437; doi:10.2196/48146)

**Multimedia Appendix 5: Comparison of chosen equation and reference material property study**

For FEA models that based their modelling on one of the top source articles, using an equation (as opposed to tabulated data), the performance of this equation was directly compared to the source data.

For models using a linear elastic equation, the Young’s modulus was used as the gradient of the stress-strain curve. For models using a hyper-elastic equation, a 3x3 element cube was created in ABAQUS (Dassault Systèmes, Paris) (Supplementary Data 4, 4.1). The cube was stretched uniaxially, with no constraint applied in the orthogonal directions, linearly increasing the nominal strain in increments of 0.05 to a maximum of 0.5. The outputs from this model were the applied true stress as a function of the applied true strain.

**5.1 Hyper-elastic modelling method** using ABAQUS (Dassault Systèmes, Paris). Left: Side view of uniaxial load test setup. Right: Typical response to uniaxial loading.


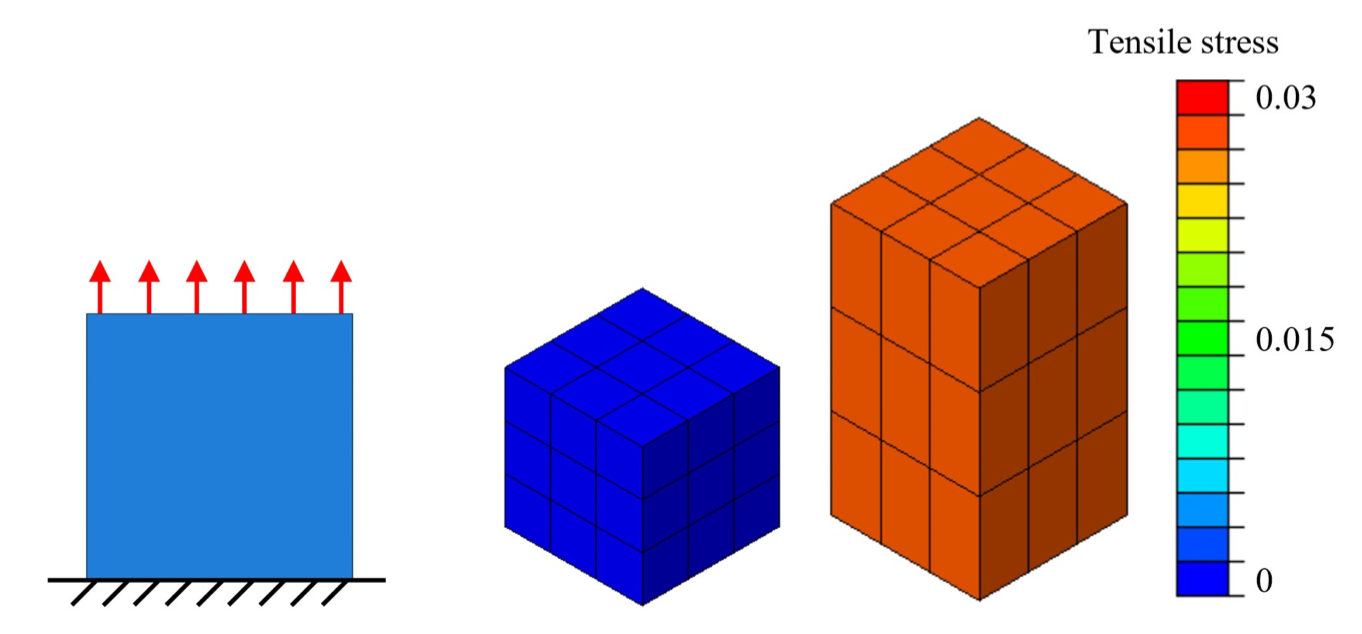


**5.2 Grey Matter**: Comparison of grey matter material experimental sources (dashed) and constitutive material models (solid). [A] Stress-strain behaviour of grey matter from shortlisted experimental sources and [B1-3] Stress-strain behaviour of FEA material models using a shortlisted source, alongside that experimental source. Plots are true stress versus true strain. For a nominal strain of 0.5 the true strain is ln(1.5)=0.405.


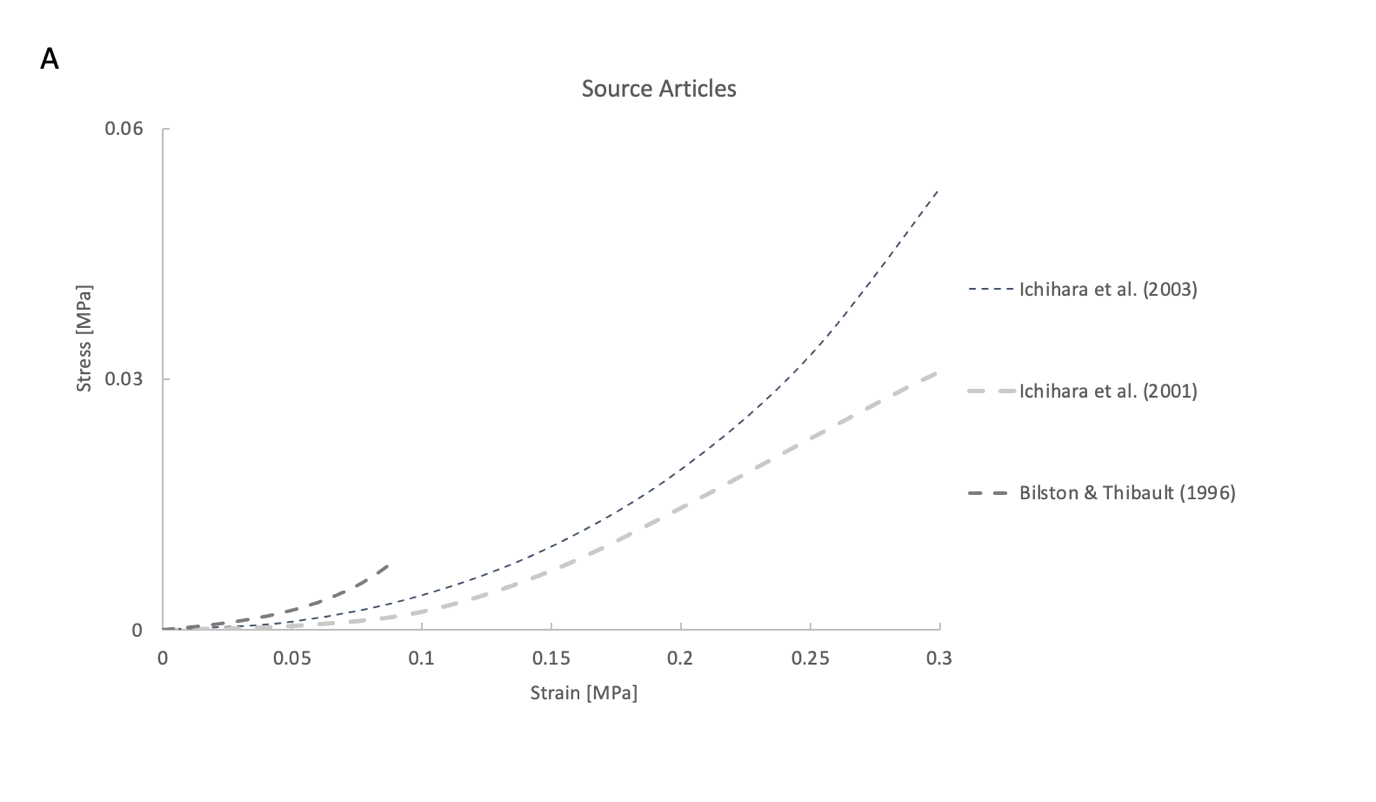


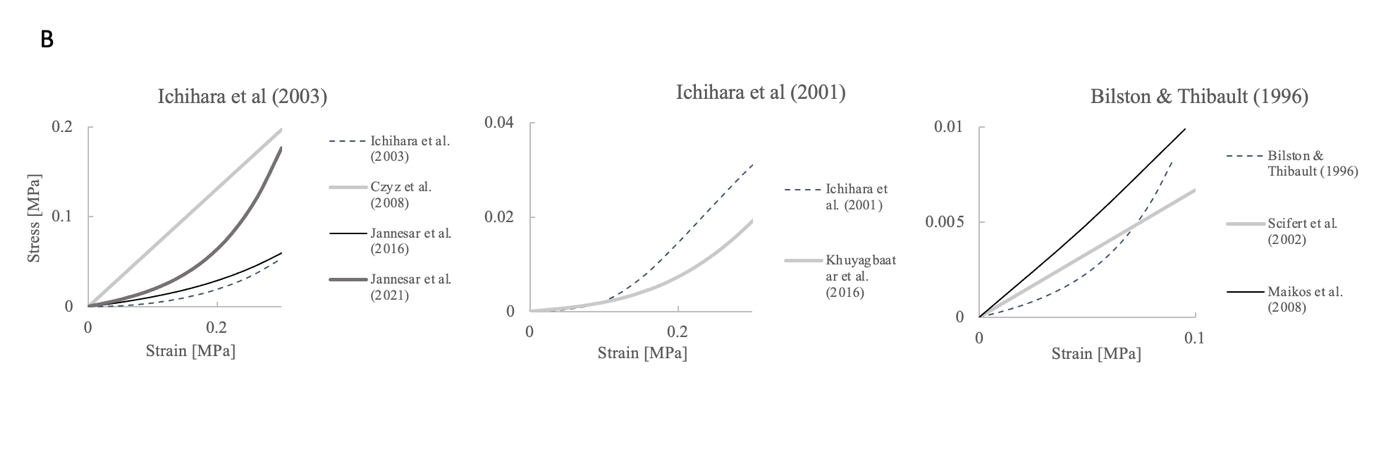


**5.3 White Matter**: Comparison of white matter material sources (dashed) and models (solid). [A] Behaviour of white matter from shortlisted sources [B1-3] Behaviour of FEA material models using a shortlisted source.


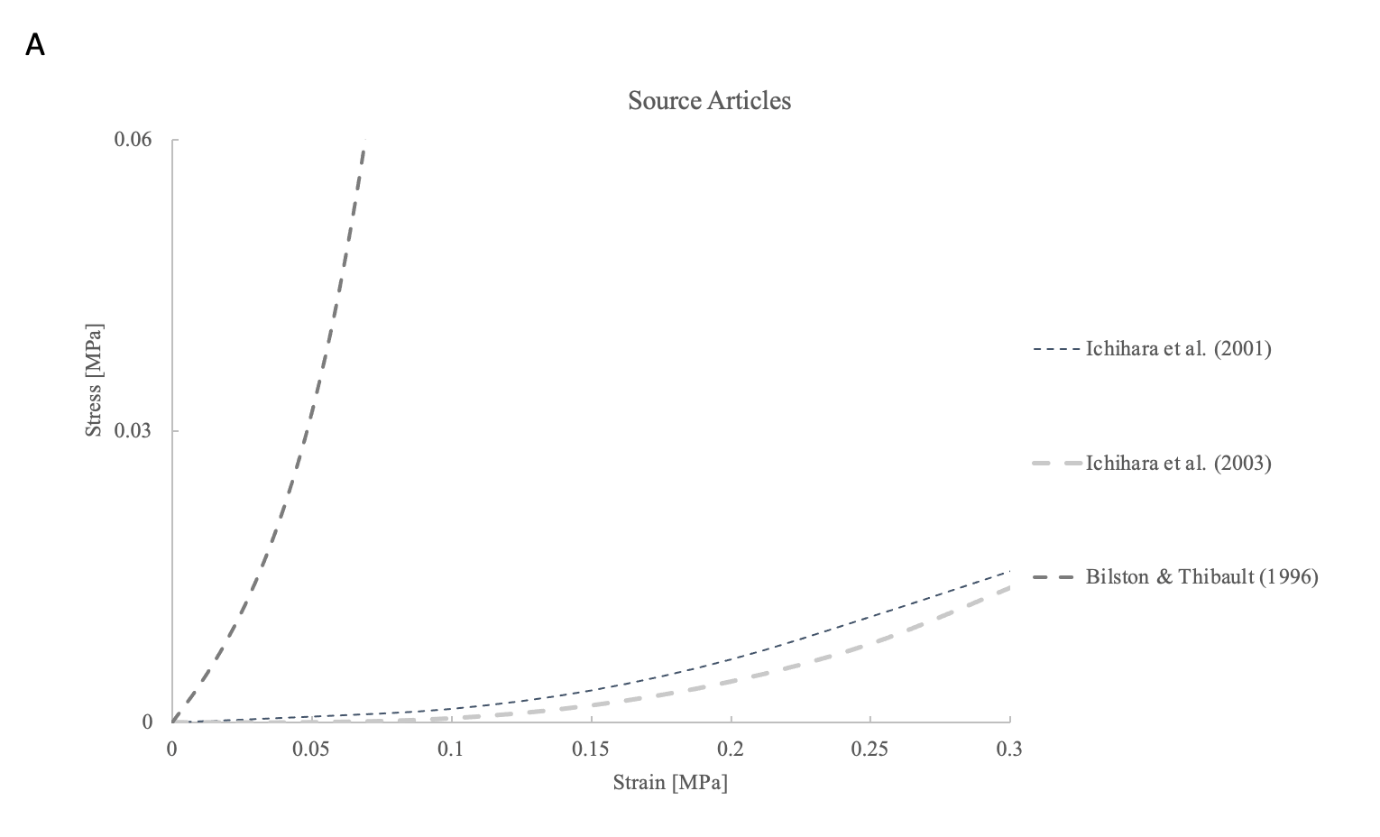


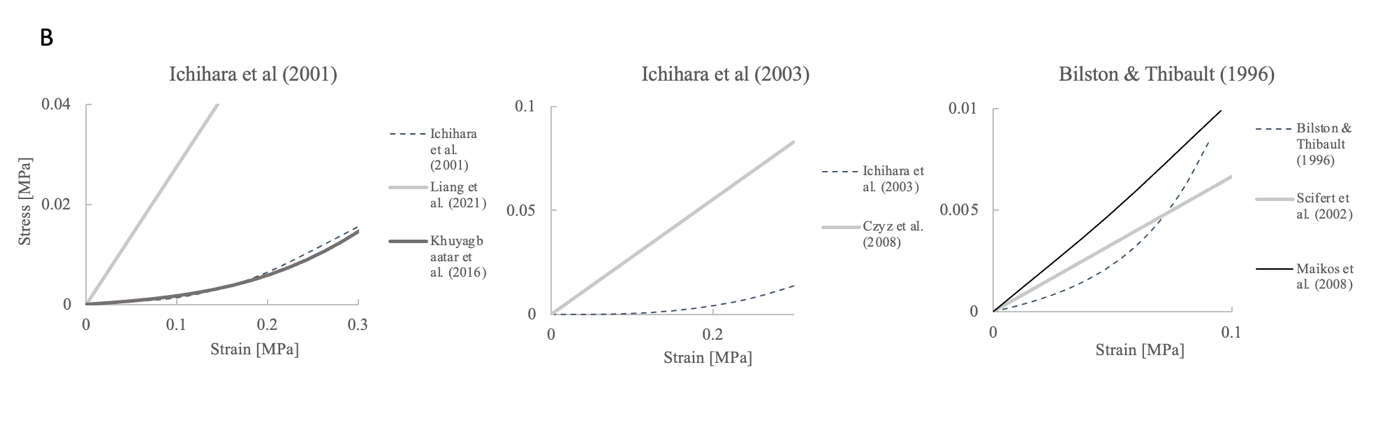


**5.3 Pia**: Comparison of pia material sources (dashed) and models (solid). [A] Behaviour of pia mater from shortlisted sources [B1-2] Behaviour of FEA material models using a shortlisted source.


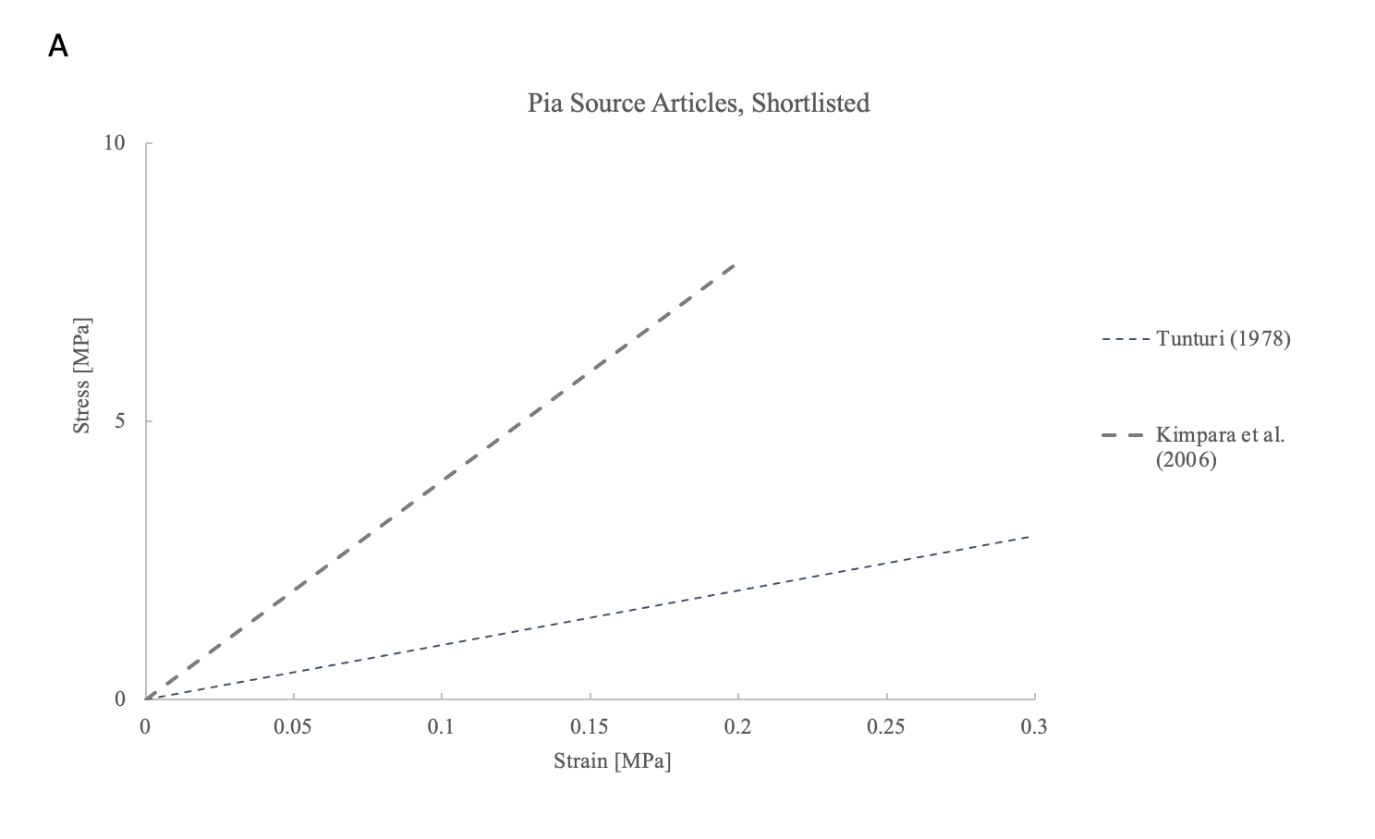


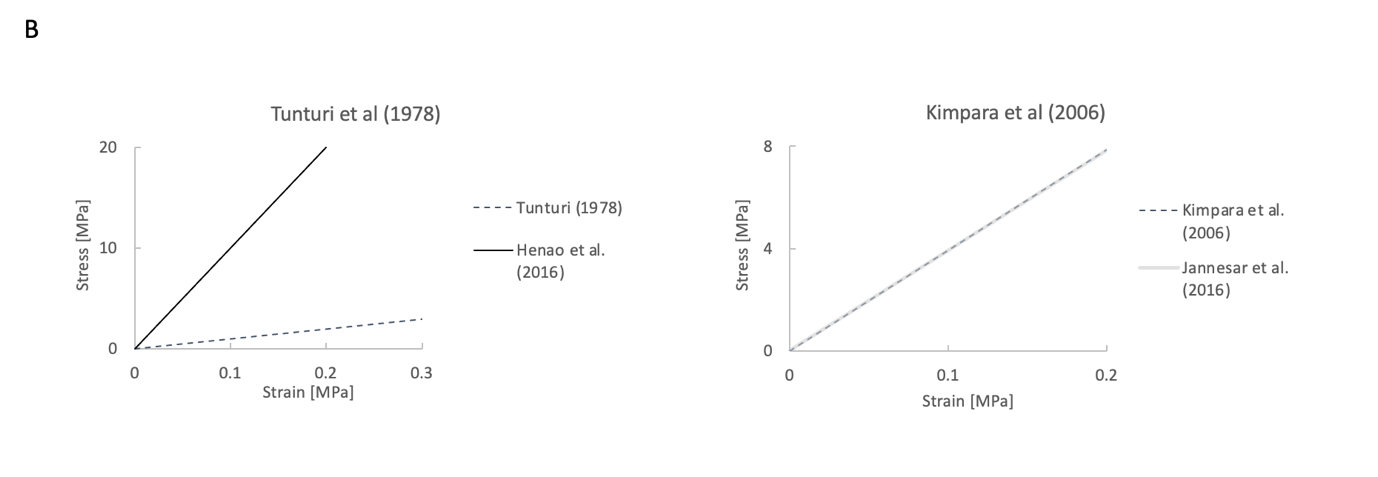


**5.4 Dura**: Comparison of source articles (dashed) and models (solid). [A] Behaviour of dura mater from shortlisted sources [B1-2] Behaviour of FEA material models using a shortlisted source.


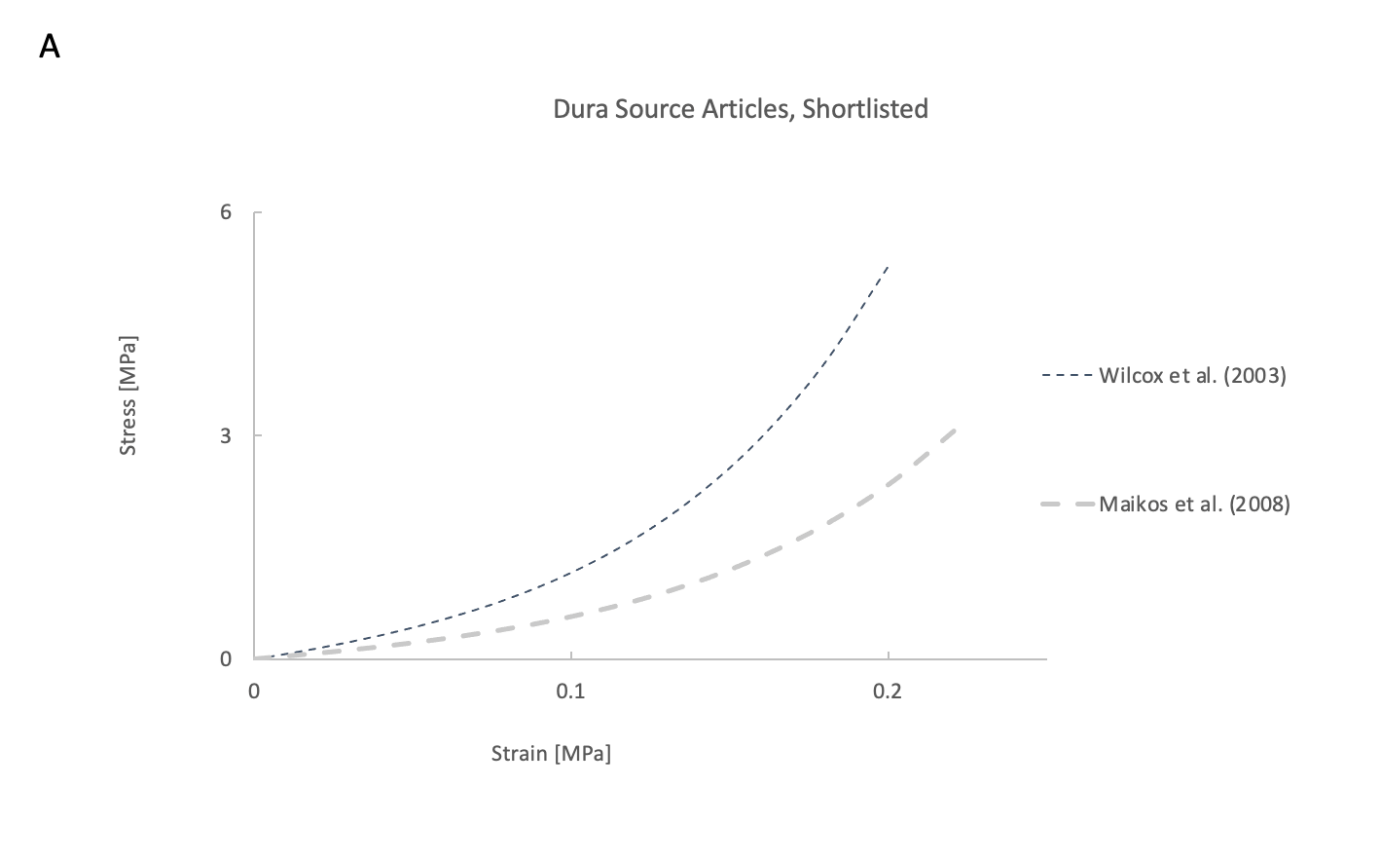


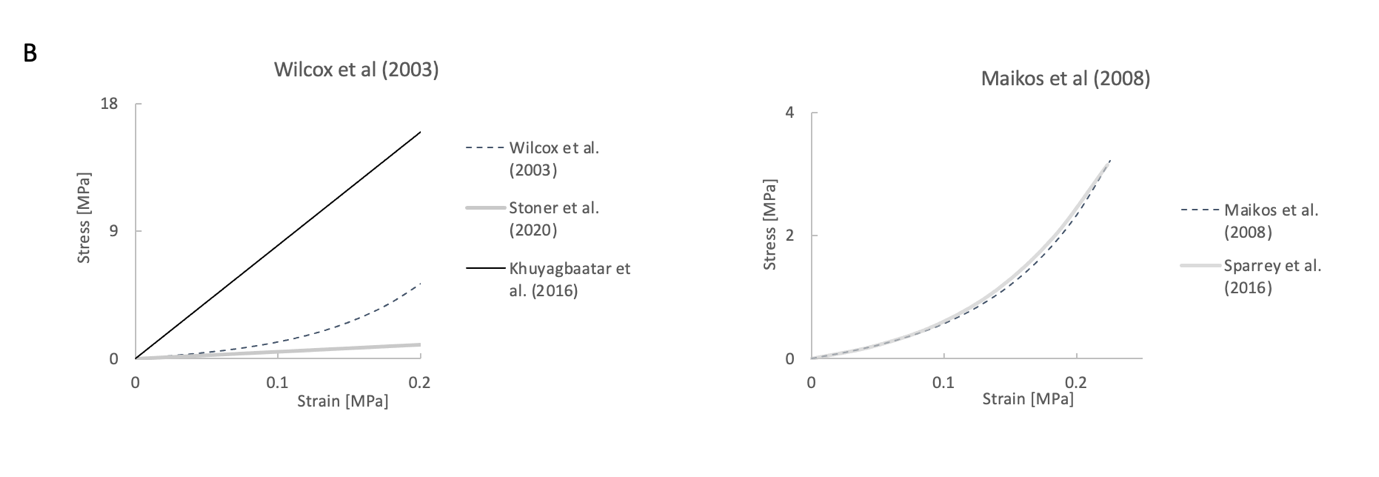

Supplement: Multimedia Appendix 5 [file biomedeng_v9i1e48146_app5.docx]
