# Supplementary material for: Finite Element Analysis for Degenerative Cervical Myelopathy: Scoping Review of the Current Findings and Design Approaches, Including Recommendations on the Choice of Material Properties
Source: JMIR Biomed Eng. 2024 Mar 28;9:e48146. doi: 10.2196/48146 (PMC11041437; doi:10.2196/48146)
Supplement: Multimedia Appendix 6 [file biomedeng_v9i1e48146_app6.docx]

**Multimedia Appendix 6: Material Properties of Other Anatomical Elements**

Identified source articles per anatomical element. The study, including DOI (Digital Object Identifier) link, and some particulars taken from the quality assessment (Supplementary Data 2) are displayed in the Tables. ‘Direct Reference’ indicates how popular this reference was, by displaying the proportion of FEA models which included this anatomical element and cited this reference. The rating refers to the overall judgement of the authors, in terms of shortlisting for a DCM FEA model.

For each anatomical element, apart from the spinal cord which is Figure 2, network diagrams are displayed below the tables. These trace the original FEA models, as either a Star, Square, Diamond or Triangle (representing their material law choice), to the primary source article (grey or black dot). An intermediate article, i.e. one that did not include primary experimental data is pale grey.

**6.1 Spinal Cord**

| **Study** | **Tissue Type** | **Aged** | **Spinal Region** | **Quality** | **Direct Reference** | **Rating** |
| --- | --- | --- | --- | --- | --- | --- |
| [Bilston 1996](http://www.doi.org/10.1007/BF02770996) | Human | Y | Cervical | High | 10% | 1 |
| [Ichihara 2003](http://www.doi.org/10.3171/spi.2003.99.3.0278) | Bovine | N | Whole | High | 17% | 1 |
| [Ichihara 2001](https://doi.org/10.1089/08977150151071053) | Bovine | N | Cervical | High | 38% | 1 |
| [Sparrey 2011](http://www.doi.org/10.1016/j.jbiomech.2011.01.035) | Porcine | N | Thoracic | Mod | 2% | 2 |
| [Ramo 2001](http://www.doi.org/10.1016/j.actbio.2018.05.045) | Murine | N | Cervical | High | 2% | 2 |
| [Chang 1988](http://www.doi.org/10.1115/1.3108415) | Feline | N | Thoracolumbar | High | 2% | 2 |
| [Hung 1982](http://www.doi.org/10.1016/0090-3019(82)90284-1) | Feline | N | Thoracic | Mod | 10% | 2 |
| [Hung 1981](http://www.doi.org/10.1115/1.3138244) | Canine | N | Thoracic | High | 2% | 2 |
| [Tunturi 1978](http://www.doi.org/10.3171/jns.1978.48.6.0975) | Canine | N | Thoracic | Mod | 2% | 2 |
| [Fiford 2005](http://www.doi.org/10.1016/j.jbiomech.2004.07.009) | Rodent | N | Whole | Mod | 2% | 3 |
| [Clarke 2009](http://www.doi.org/10.1016/j.jbiomech.2009.04.008) | Rodent | N | Whole | High | 0% | 3 |
| [Ozawa 2004](http://www.doi.org/10.3171/spi.2004.1.1.0122) | Rabbit | ? | Cervical | Mod | 5% | 3 |
| [Ozawa 2001](http://www.doi.org/10.3171/spi.2001.95.2.0221) | Rabbit | ? | Cervical | Mod | 2% | 3 |
| [Shetye 2013](http://www.doi.org/10.1016/j.actbio.2013.10.038) | Porcine | N | Lumbar | Mod | 2% | 3 |

**6.2 Pia**

| **Study** | **Tissue Type** | **Aged** | **Spinal Region** | **Quality** | **Direct Reference** | **Rating** |
| --- | --- | --- | --- | --- | --- | --- |
| [Kimpara 2006](https://pubmed.ncbi.nlm.nih.gov/17311175/) | Porcine | ? | Cervical | Mod | 38% | 2 |
| [Tunturi 1978](http://www.doi.org/10.3171/jns.1978.48.6.0975) | Canine | N | Thoracic | Mod | 4% | 2 |
| [Ozawa 2004](http://www.doi.org/10.3171/spi.2004.1.1.0122) | Rabbit | ? | Cervical | High | 19% | 3 |
| [Jin 2010](http://www.doi.org/10.1016/j.jbiomech.2010.09.035) | Bovine | N | Brain | High | 0% | 3 |


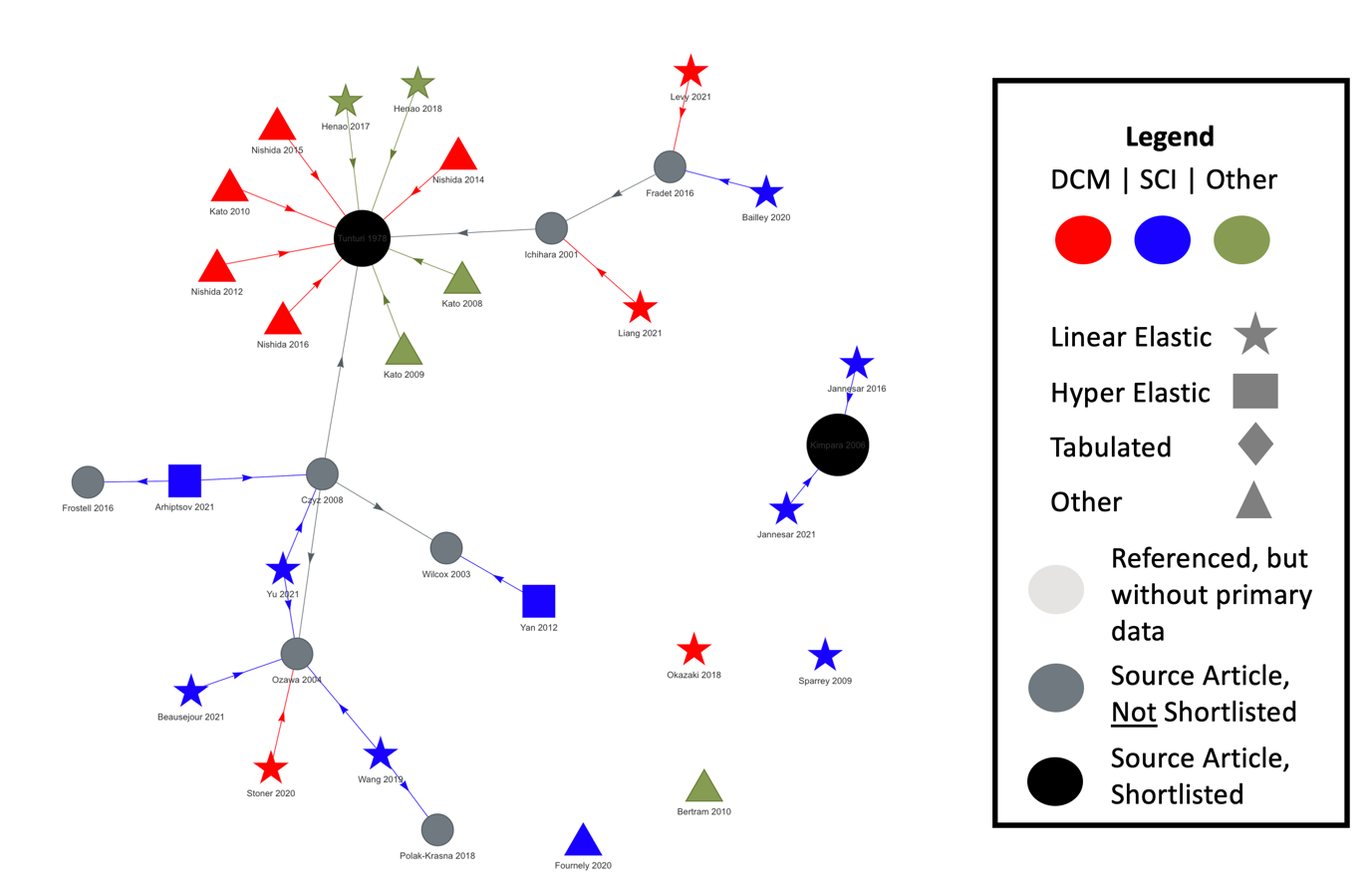


**6.3 Dura**

| **Study** | **Tissue Type** | **Aged** | **Spinal Region** | **Quality** | **Direct Reference** | **Rating** |
| --- | --- | --- | --- | --- | --- | --- |
| [Hong 2011](http://www.doi.org/10.1016/j.spinee.2011.11.001) | Human | Y | Thoracolumbar | High | 4% | 1 |
| [Zarzur 1996](http://www.doi.org/10.1590/s0004-282x1996000300015) | Human | N | Lumbar | Mod | 4% | 1 |
| [Persson 2020](http://www.doi.org/10.1007/s10439-010-9924-6) | Bovine | N | Whole | High | 27% | 1 |
| [Wilcox 2003](http://www.doi.org/10.1007/s10776-003-0644-9) | Bovine | ? | Whole | Mod | 19% | 1 |
| [Tunituri 1977](http://www.doi.org/10.3171/jns.1977.47.3.0391) | Canine | ? | Thoracic | Mod | 4% | 2 |
| [Runza 1999](http://www.doi.org/10.1097/00000539-199906000-00022) | Bovine | N | Lumbar | Mod | 4% | 2 |
| [Maikos 2008b](http://www.doi.org/10.1089/neu.2007.0348) | Rodent | N | Whole | High | 0% | 3 |
| [Galford 1970](http://www.doi.org/10.1016/0021-9290(70)90007-2) | Monkey | ? | Brain | Mod | 0% | 3 |
| [van Noort 1981](http://www.doi.org/10.1016/0142-9612(81)90086-7) | Human | ? | ? | Low | 8% | 3 |


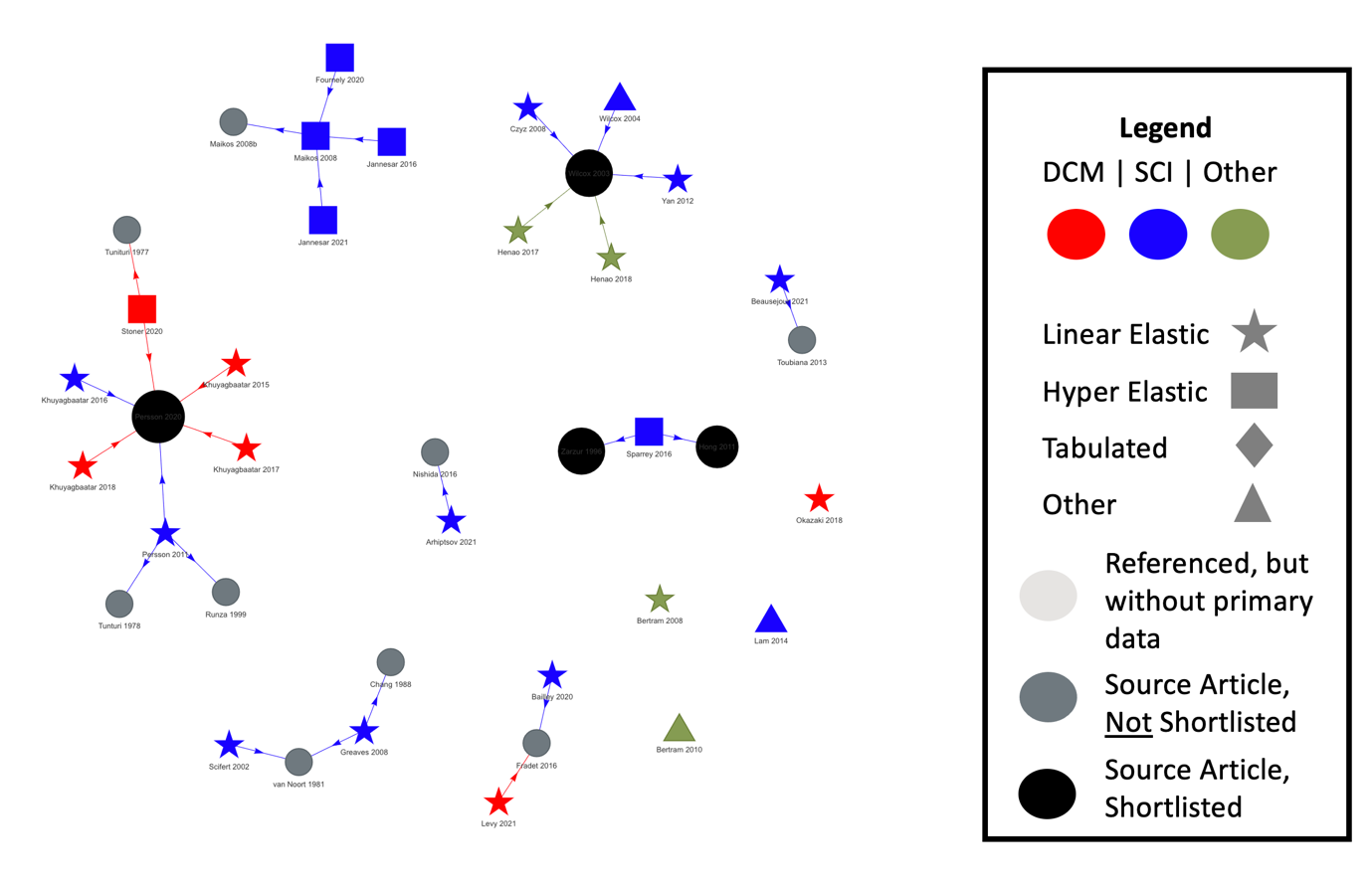


**6.4 Dentate Ligament**

| **Study** | **Tissue Type** | **Aged** | **Spinal Region** | **Quality** | **Direct Reference** | **Rating** |
| --- | --- | --- | --- | --- | --- | --- |
| [Tubbs 2001](http://www.doi.org/10.3171/spi.2001.94.2.0271) | Human | Y | Cervical | High | 0% | 1 |
|  | Canine | N | Thoracic | Mod | 15% | 2 |


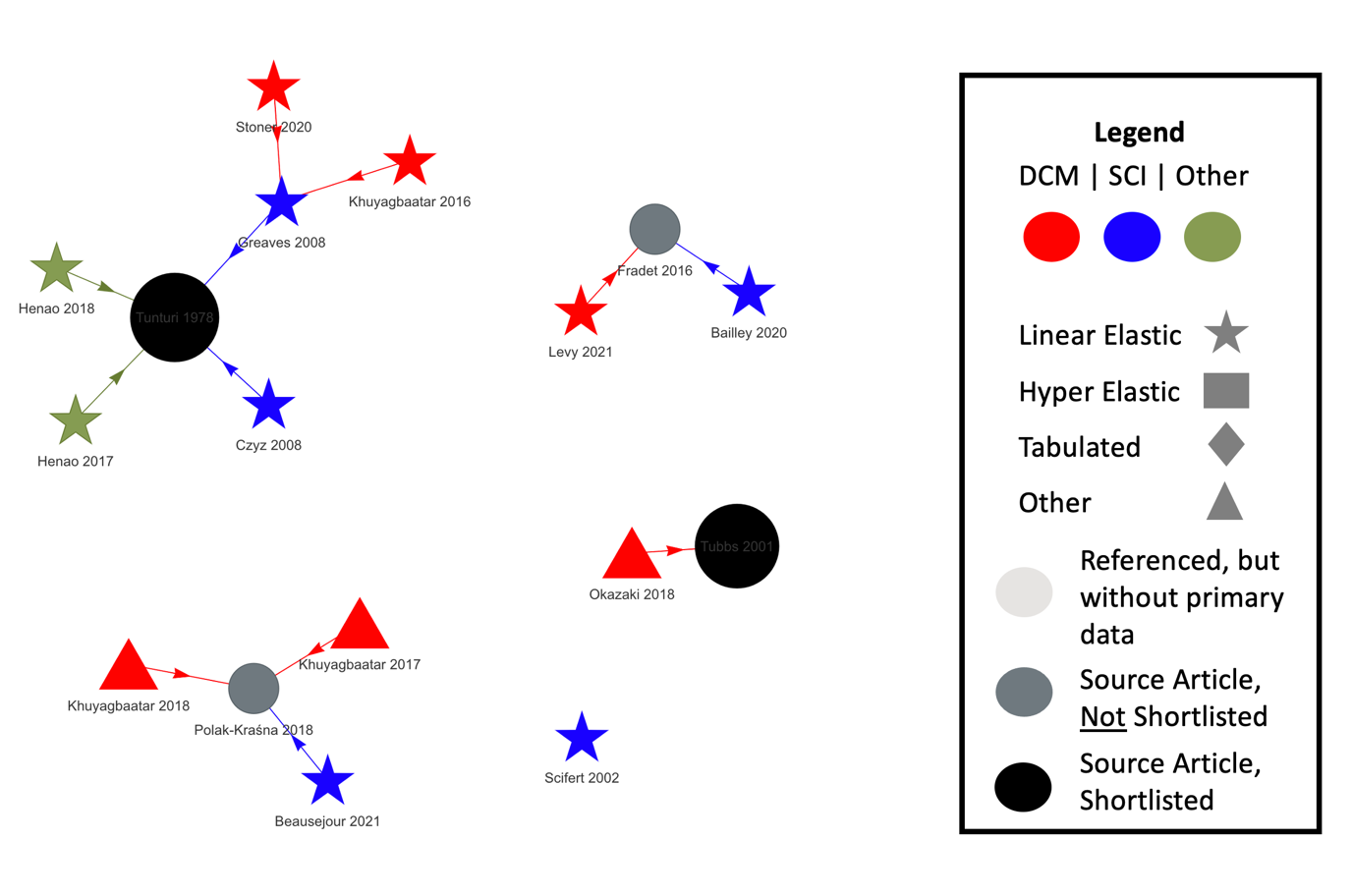


**6.5 CSF**

| **Study** | **Tissue Type** | **Aged** | **Spinal Region** | **Quality** | **Direct Reference** | **Rating** |
| --- | --- | --- | --- | --- | --- | --- |
| [Bloomfield 1998](file:///C:\temp\10.1159\000028659) | Human | ? | N/A | Mod | 35% | 1 |
| [Brydon 1995](file:///C:\temp\10.1080\02688699550040927) | Human | N | N/A | High | 24% | 1 |
| [Persson 2011](http://www.doi.org/10.1089/neu.2010.1332) | Bovine | N | Thoracic | High | 6% | 1 |
| [Shreiber 1997](http://www.doi.org/10.4271/973335) | Rodent | N | Brain | Mod | 6% | 2 |
| [Chafi 2009](file:///C:\temp\10.1243\09544119JEIM631) | Human | N | Brain | High | 6% | 2 |


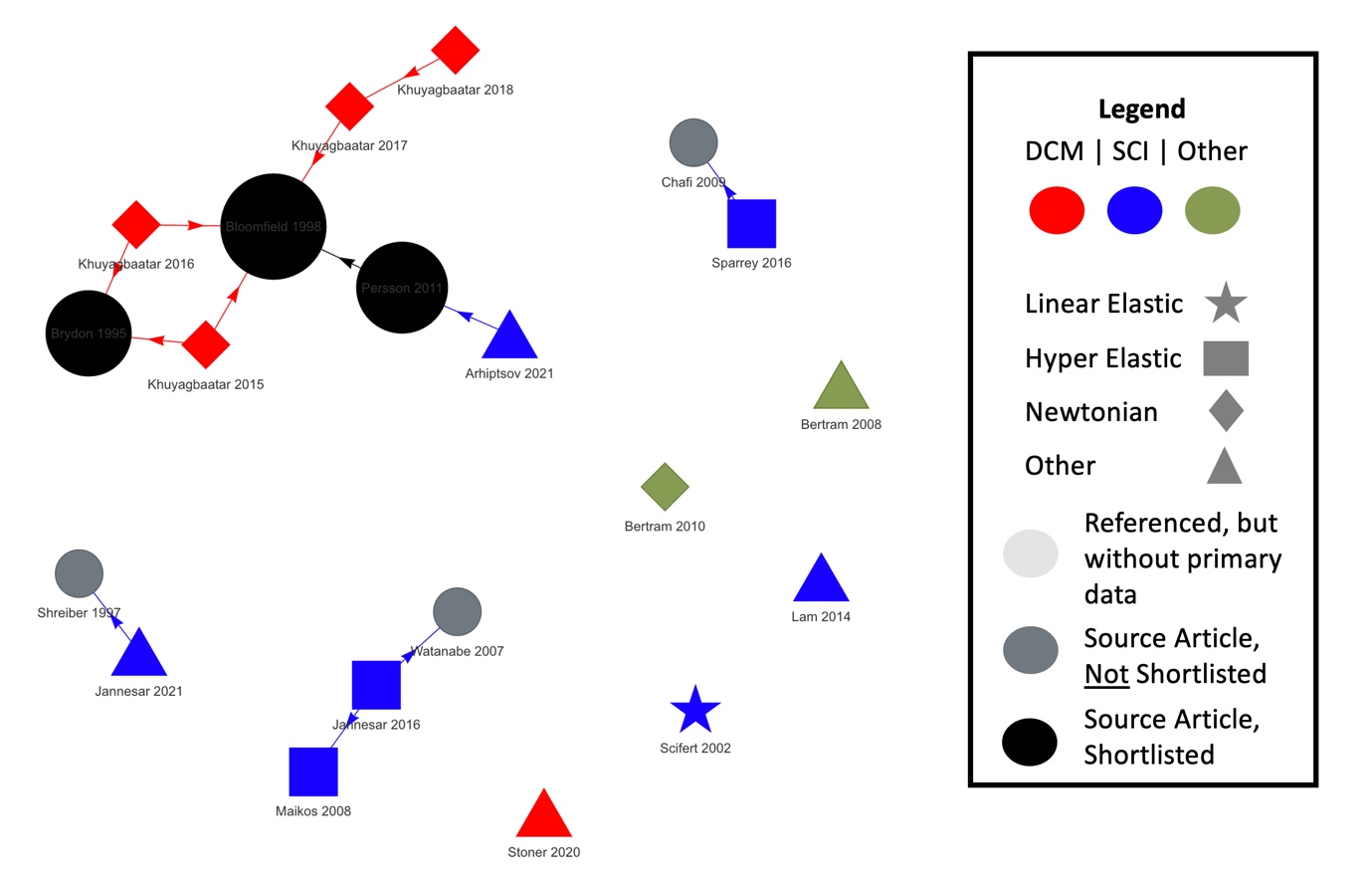


**6.6 Posterior Longitudinal Ligament and Ligamentum Flavum**

| **Study** | **Tissue Type** | **Aged** | **Spinal Region** | **Quality** | **Direct Reference** | **Rating** |
| --- | --- | --- | --- | --- | --- | --- |
| [Przybylski 1996](file:///C:\temp\10.1002\jor.1100140623) | Human | Y | Cervical | High | 17% | 1 |
| [Yoganandan 2000](file:///C:\temp\10.1115\1.1322034) | Human | Y | Cervical | High | 17% | 1 |
| [Yoganandan 1989](http://www.doi.org/10.1097/00007632-198910000-00013) | Human | Y | Cervical | High | 17% | 1 |
| [Mattucci 2012](http://www.doi.org/10.1016/j.jmbbm.2012.02.004) | Human | N | Cervical | High | 17% | 2 |
| [Mattucci 2014](http://www.doi.org/10.1016/j.jmbbm.2014.09.023) | Human | N | Cervical | High | 0% | 2 |
| [Tkaczuk 1968](http://www.doi.org/10.3109/ort.1968.39.suppl-115.01) | Human | Y | Lumbar | High | 17% | 2 |


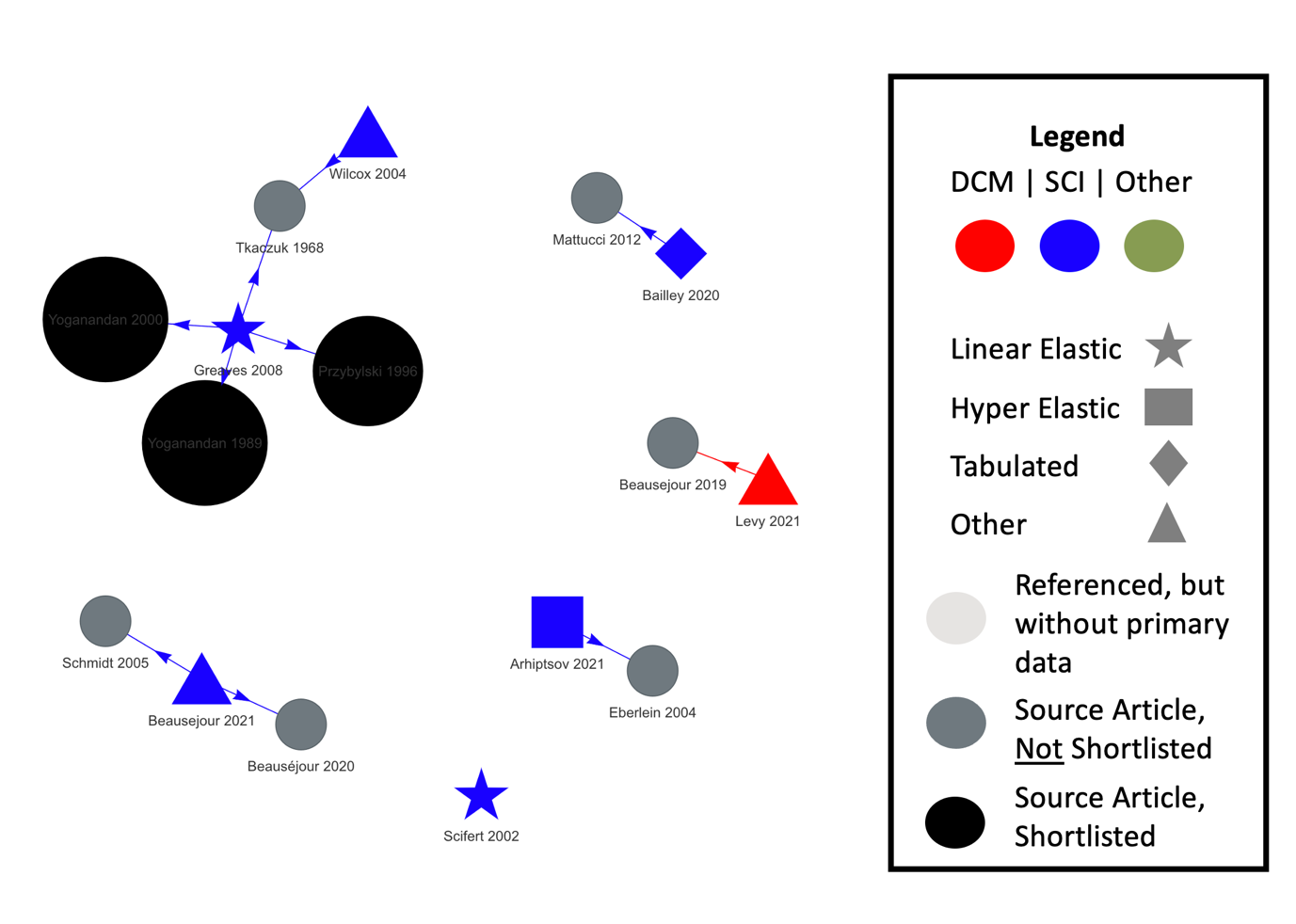


**6.7 Spinal Root**

| **Study** | **Tissue Type** | **Aged** | **Spinal Region** | **Quality** | **Direct Reference** | **Rating** |
| --- | --- | --- | --- | --- | --- | --- |
| [Kulkarni 2007](http://www.doi.org/10.1016/j.jneumeth.2007.06.025) | Rodent | N | Lumbar | High | 43% | 2 |
| [Singh 2005](http://www.doi.org/10.1016/j.jbiomech.2005.04.023) | Rodent | N | Lumbar | High | 29% | 2 |


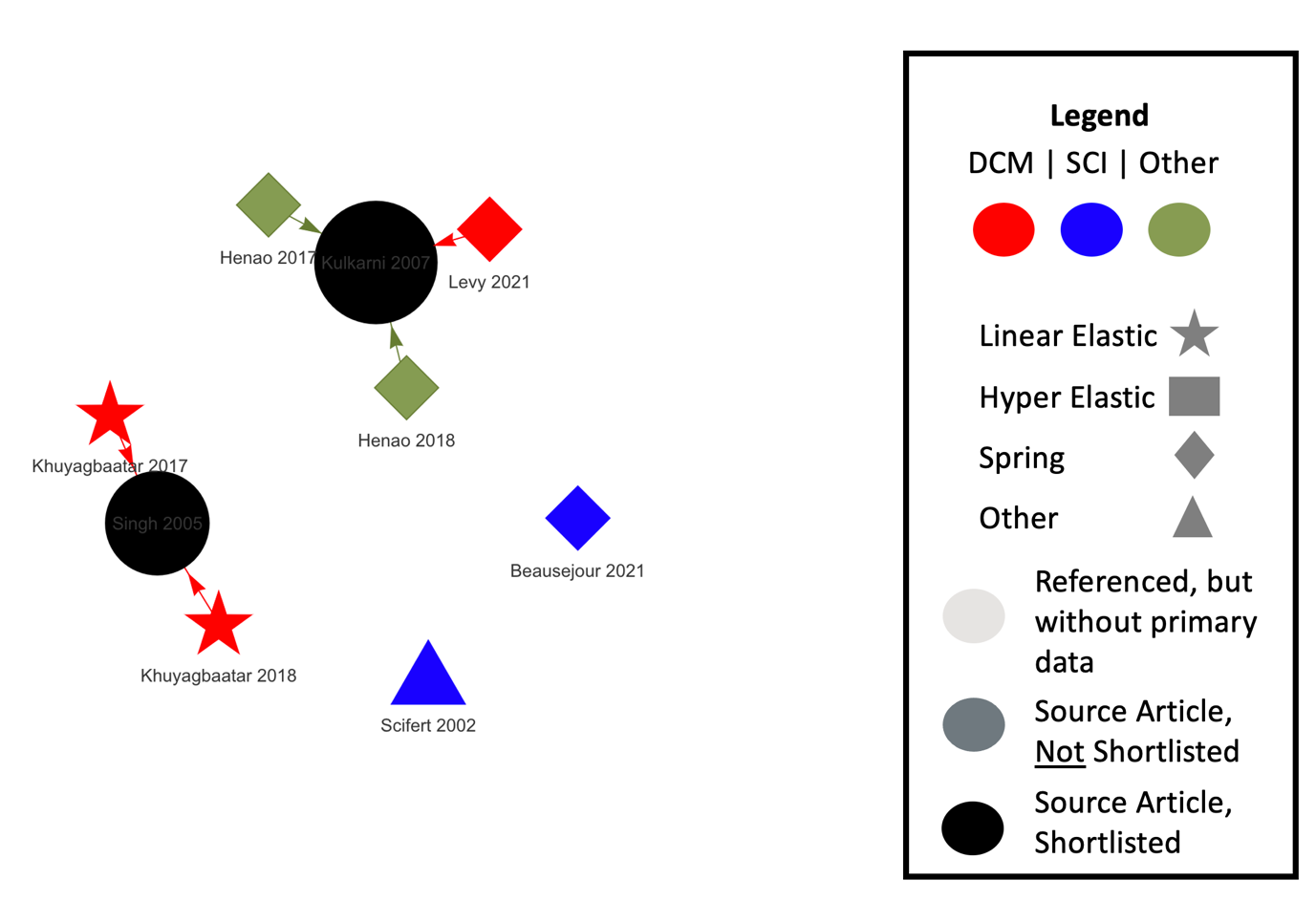


**6.8 Intervertebral Disc**

| **Study** | **Tissue Type** | **Aged** | **Spinal Region** | **Quality** | **Direct Reference** | **Rating** |
| --- | --- | --- | --- | --- | --- | --- |
| [Spiker 1986](http://www.doi.org/10.1115/1.3138575) | Human | Y | Lumbar | High | 8% | 1 |
| [Schmidt 2005](http://www.doi.org/10.1016/j.clinbiomech.2005.12.001) | Human | ? | Lumbar | Mod | 8% | 2 |
| [Brown 1957](https://pubmed.ncbi.nlm.nih.gov/13475413/) | Human | ? | Lumbar | Low | 8% | 2 |
